# Applications of M_*x*_Se_*y*_ (M = Fe, Co, Ni) and Their Composites in Electrochemical Energy Storage and Conversion

**DOI:** 10.1007/s40820-019-0272-2

**Published:** 2019-05-15

**Authors:** Huijie Zhou, Xiaxia Li, Yan Li, Mingbo Zheng, Huan Pang

**Affiliations:** School of Chemistry and Chemical Engineering, Guangling College, Yangzhou University, Yangzhou, 225009 Jiangsu People’s Republic of China

**Keywords:** M_*x*_Se_*y*_, Battery, Supercapacitor, Electrocatalysis

## Abstract

Transition-metal selenides (M_*x*_Se_*y*_, M = Fe, Co, Ni) and their composites exhibit good storage capacities for sodium and lithium ions and occupy a unique position in research on sodium-ion and lithium-ion batteries. M_*x*_Se_*y*_ and their composites are used as active materials to improve catalytic activity. However, low electrical conductivity, poor cycle stability, and low rate performance severely limit their applications. This review provides a comprehensive introduction to and understanding of the current research progress of M_*x*_Se_*y*_ and their composites. Moreover, this review proposes a broader research platform for these materials, including various bioelectrocatalytic performance tests, lithium–sulfur batteries, and fuel cells. The synthesis method and related mechanisms of M_*x*_Se_*y*_ and their composites are reviewed, and the effects of material morphologies on their electrochemical performance are discussed. The advantages and disadvantages of M_*x*_Se_*y*_ and their composites as well as possible strategies for improving the storage and conversion of electrochemical energy are also summarized.

## Highlights

- The electrochemical properties of M_*x*_Se_*y*_ (M = Fe, Co, Ni) and their Composites have been discussed.
- The synthetic methods and morphologies have been summarized.
- The future directions and application prospect of M_*x*_Se_*y*_ (M = Fe, Co, Ni) and their composites are given.

## Graphical abstract

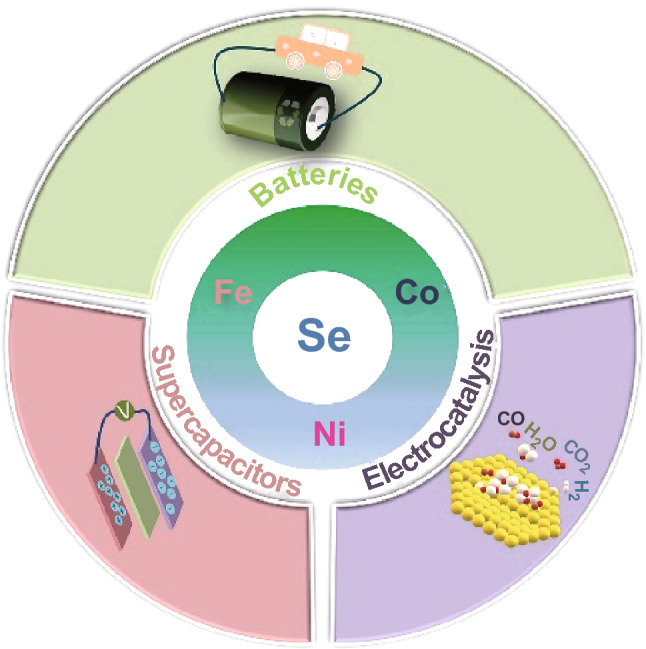

## Introduction

As the main form of energy for storage, output, and conversion, electric energy is widely used in all aspects of industrial production and life. It has become the driving force for the development of science and technology [1, 2]. Therefore, developing an electrochemical power source with high power density, large storage capacity, high energy density, long service life, and low cost is of significant importance [3–6]. Among the recent studies on electrochemical energy storage and conversion, many have reported on transition-metal sulfides, such as MoS_2_ [7–9], VS_2_ [10–12], CoS_2_ [13–16], FeS_2_ [17–20], and SnS_2_ [21–24], especially in the field of electrochemical energy storage and conversion. Because selenium and sulfur belong to the same main group, there are certain similarities in their physical and chemical properties [25]. Therefore, transition-metal selenides (M_*x*_Se_*y*_) and their composites continue to be the focus of research on electrochemical energy storage and conversion.

In recent years, significant advances have been made in M_*x*_Se_*y*_ and their composites for their application in electrochemical energy storage and conversion. The ordered nanoarray structure of M_*x*_Se_*y*_ can eliminate the disadvantages posed by traditional electrode preparation technologies, shorten the diffusion path of ions, expand the contact area between the electrode material and electrolyte, avoid the aggregation of the electrode material in a Faraday reaction, and increase the load of the active material on the collector [1, 26, 27]. Moreover, because of the inherent metallic properties of multicomponent selenides, synergistic effect among polymetallic ions, superhydrophilicity of the material surface, unique honeycomb array, and multistructural robustness, M_*x*_Se_*y*_ and their composites have broad application prospects [28–30]. The good cycle performance and ideal conductivity of M_*x*_Se_*y*_ make it an electrode material with wide-ranging application prospects.

**Fig. 1:**
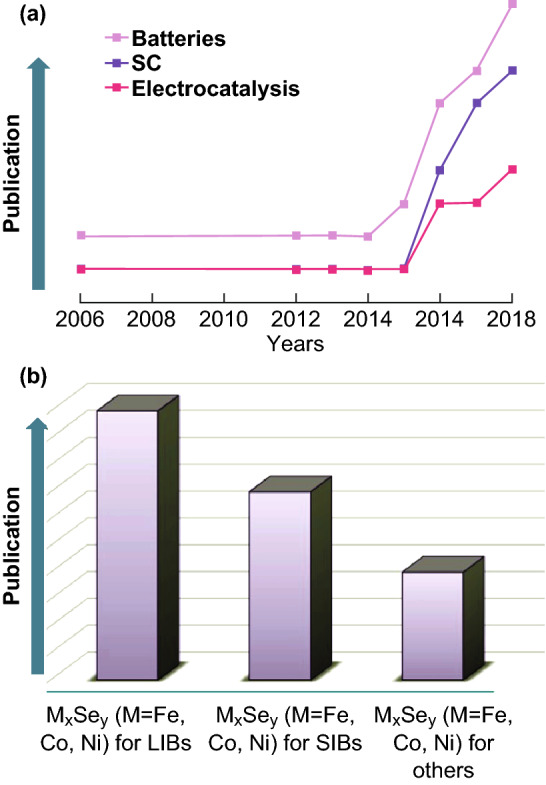
**a** Research trends of transition-metal selenides in electrochemical energy storage (batteries, SCs) and conversion (electrocatalysis) from 2006 to 2018. **b** Use of transition-metal selenides in various types of batteries (LIBs, SIBs, and other batteries)

In this review, we summarize the uses of M_*x*_Se_*y*_ (M = Fe, Co, Ni) and their composites in electrochemical energy storage and conversion, such as battery and supercapacitor (SC) applications. M_*x*_Se_*y*_ and their composites are also widely used in electrochemical energy conversion applications such as the oxygen evolution reaction (OER), oxygen reduction reaction (ORR), and hydrogen evolution reaction (HER). Moreover, M_*x*_Se_*y*_ (M = Fe, Co, Ni) and their composites find applications in other electrochemical energy fields. As shown in Fig. 1a, we review the related literature from 2006 to 2018 to summarize the research and application trends of M_*x*_Se_*y*_ (M = Fe, Co, Ni) and their composites in batteries, SCs, and electrocatalysis. The related research has increased year after year, especially in the field of batteries. Additionally, as shown in Fig. 1b, we detail their applications in various subareas of batteries, such as lithium-ion batteries (LIBs), sodium-ion batteries (SIBs), and others. As can be seen, these materials are widely studied for battery applications, especially in LIBs.

## Batteries

Rechargeable batteries have become one of the most attractive energy storage devices because they offer environmental protection, high storage efficiency, low maintenance cost, good cycle stability, and satisfactory energy and power performance [31]. The charge state of any rechargeable battery, regardless of its chemical nature, is a necessary parameter for battery management systems of hybrid electric vehicles [32]. Furthermore, electrode materials play an important role in improving the electrochemical performance of batteries [33]. Electrode materials used in the most advanced single metal-ion batteries (such as LIBs and SIBs) offer the advantages of environmental protection, high storage efficiency, low maintenance cost, good cycle stability, and good power performance [34]. Therefore, applications of M_*x*_Se_*y*_ in the fields of rechargeable batteries, SCs, and catalysts have attracted increasing attention.

### Lithium-Ion Battery

Rechargeable LIBs are electrochemical energy storage devices consisting of two electrodes connected by an electrolyte and a separation membrane [35]. LIBs, with their highest energy density and longer cycle life, dominate the rechargeable, portable electronics, and electric vehicle markets [36–38]. However, LIBs have many drawbacks. For example, their constituents are toxic and combustible and cause other safety-related problems [10, 11]. Therefore, owing to their excellent properties, TiSe_2_, ZrSe_2_, NbSe_2_, Te_2_Se, Mo_6_Se_6_, and VSe have been widely used as rechargeable Li battery energy storage materials.

#### Cobalt Selenium

**Fig. 2:**
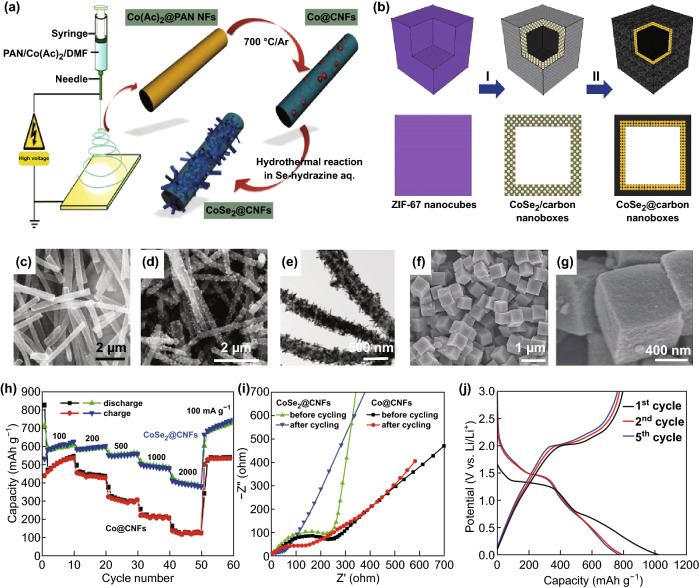
**a** Schematic diagram of the synthesis of Co@CNF and CoSe_2_@CNF. **b** Schematic diagram of CoSe@CNF formation process. **c** SEM image of Co@CNFs. **d** SEM image of CoSe_2_@CNFs. **e** TEM image of CoSe_2_@CNFs. **f, g** Images showing annealing of a ZIF-67 nm cube and Se powder for 2 h and synthetic CoSe_2_/CNF FE-SEM under a nitrogen atmosphere at 350 °C. **h** Rate performance at different current densities. **i** The electrochemical performance of CoSe@CNF and CoSe_2_@CNF electrodes before and after cycling in the Nyquist diagram. **j** Discharge and charge voltage curves at a current density of 0.2 A g^−1^. Adapted from Refs. [39, 40] with permission. Copyrights: 2016 (2017), Wiley-VCH

As an important functional material, M_*x*_Se_*y*_ has been widely studied for its use in energy storage. Recently, Wang et al. prepared CoSe_2_@carbon nanotubes (CNFs) (Fig. 2d, e) by electrospinning and subsequent annealing using encapsulated Co nanoparticles (Co@CNFs, Fig. 2c) to synthesize porous graphite CNFs (Fig. 2a). When used as a negative electrode material for LIBs, CoSe_2_@CNF has excellent Li storage properties and a high reversible capacity of 1405 mAh g^−1^ after 300 cycles at a current density of 200 mA g^−1^ (Fig. 2h, i) [39]. The hollow structure with a special shell shape is a popular focus in studies on electrochemical energy storage applications. Starting from metal–organic frameworks (MOFs), Hu et al. [40] reported on the generation of CoSe@CNFs using zeolitic imidazolate framework (ZIF-67) nanomicrospheres and the Se powder as the precursor (Fig. 2b). In the synthesis process, CoSe_2_ nanoparticles were uniformly distributed in the carbon shell at a low annealing temperature. After further annealing at a high temperature, CoSe_2_ nanoparticles were further chemically transformed into CoSe@CNFs (Fig. 2f, g). The CoSe@CNF composite had initial charge and discharge capacities of 796 and 1016 mAh g^−1^, respectively, at a current density of 0.2 A g^−1^, and its initial coulombic efficiency was 78.3% (Fig. 2j). The slow redox kinetics and dissolution of polyselenides can cause severe degradation of the Li–Se battery capacity, thereby limiting their practical applications to some extent. Later, Li et al. [41] used ZIF-67 as a precursor to embed advanced CoSe nanoparticles in a novel porous carbon polymer to produce a new CoSe@PCP composite for LIBs and SIBs. CoSe@PCP not only has an outstanding lithium-ion storage performance but also a high specific capacity. In addition, it has excellent rate performance and a long life. The proposed strategy not only provides a method for synthesizing nanoscale electroactive materials and porous carbon composites with a regular morphology but also shows the considerable potential of selenide as a high-performance anode material for LIBs and silicon carbon rods. Yang et al. [42] prepared a mixed CoSe_2_N-CF/CNT superstructure composed of internal CoSe_2_ nanoparticles and external CNT-entangled N-doped carbon skeletons through ZIF-67 by a simple two-step heat treatment procedure for LIBs and SIBs. The electrochemical reactions of CoSe_2_@N-CF/CNTs for lithium are as follows:

*Lithium storage*

1 $${\text{Discharge}}\,{\text{process}}\,{\text{CoSe}}_{2} + x{\text{Li}} + x{\text{e}}^{ - } \to {\text{Li}}_{x} {\text{CoSe}}_{2}$$

2 $${\text{Li}}_{x} {\text{CoSe}}_{2} + {\text{Li}}^{ + } + {\text{e}}^{ - } \to {\text{Co}} + {\text{Li}}_{2} {\text{Se}}$$

3 $${\text{Charge}}\,{\text{process}}\,{\text{Co}} + {\text{Li}}_{2} {\text{Se}} \to {\text{Li}}_{x} {\text{CoSe}}_{2} + {\text{Li}}^{ + } + {\text{e}}^{ - }$$

4 $${\text{Li}}_{x} {\text{CoSe}}_{2} \to {\text{CoSe}}_{2} + x{\text{Li}}^{ + } + x{\text{e}}^{ - }$$

5 $${\text{Total}}\,{\text{reaction}}\,{\text{equation}}\;{\text{CoSe}}_{2} + 4{\text{Li}}^{ + } + 4{\text{e}}^{ - } \,\leftrightarrow \,{\text{Co}} + 2{\text{Li}}_{2} {\text{Se}}$$

Dual-anode materials in LIBs and SIBs exhibit excellent electrochemical performance. (They also provide a high reversible capacity of 428 mAh g^−1^ after 500 cycles of an LIB, at 0.1 A g^−1^, and capacity of 606 mAh g^−1^ after 100 cycles of an SIB.) In addition, electrochemical kinetic analysis shows that at high scan rates, tantalum capacitance contributes to the charge storage capacity, thus achieving a high rate performance of LIBs and SIBs. The effective synthesis strategy described in this work provides new insights into the high-performance dual-anode materials of LIBs and SIBs. Gao et al. [43] synthesized CoSe_2_ MoSe_2_/C HNT with high reversibility and excellent rate performance for Li^+^ storage, with high capacity retention at higher currents; this indicates that hybrid nanostructures have broad application prospects in LIBs.

**Fig. 3:**
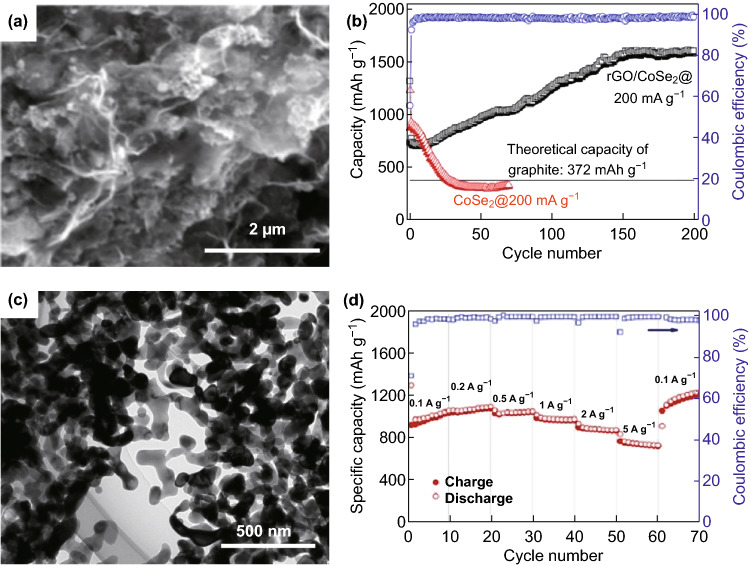
**a** SEM images of rGO/CoSe_2_ composite. **b** Cycling performance of rGO/CoSe_2_ composite. **c** TEM image of porous Cu-doped CoSe_2_. **d** Capacitances at current densities varying from 0.1 to 5.0 A g^−1^. Adapted from Refs. [44, 45] with permission. Copyrights: 2015 (2017), Wiley-VCH

The structure has a significant influence on the material properties, and the spatial structure of the synthetic material has a certain influence on the conduction of electrons. For example, Li et al. [44] synthesized a graphene/cobalt selenide (rGO/CoSe_2_) composite with pleats. Figure 3a shows that CoSe_2_ nanoparticles are tightly anchored/wrapped on rGO flakes. The rGO/CoSe_2_ composite has better cycle and electrochemical properties compared with those of pure CoSe_2_ (Fig. 3b). In this study, the innovative composite of CoSe_2_ and rGO not only reduces the structural degradation caused by volume expansion but also effectively improves the electrical conductivity of the entire electrode, thereby significantly improving the storage performance of lithium. Nevertheless, Chen et al. [45] used ZIF-67 as a precursor and the Se powder to synthesize porous copper-doped CoSe_2_ with nanoparticles interconnected by annealing (Fig. 3c). The porous material can be used as a negative electrode material for LIBs during the charge and discharge processes. It is well adapted to volume expansion, and the porous structure effectively shortens the ion and electron transport paths. This gives it a superior rate performance (Fig. 3d) and good cycle stability.

#### Nickel Selenium

Recently, scientists have synthesized electrode materials with superior properties for Li storage by means of pulsed laser deposition. Yang et al. [46] successfully synthesized the NiO–NiSe nanocomposite film by pulsed laser deposition and used it as an anode material of LIBs to explore the mechanism of the electrochemical reaction as follows:

6 $${\text{NiO}} + {\text{NiSe}} + 4{\text{Li}}^{ + } + 4{\text{e}}^{ - }\, { \leftrightarrows }\,2{\text{Ni}} + {\text{Li}}_{2} {\text{O}} + {\text{Li}}_{2} {\text{Se}}$$

**Fig. 4:**
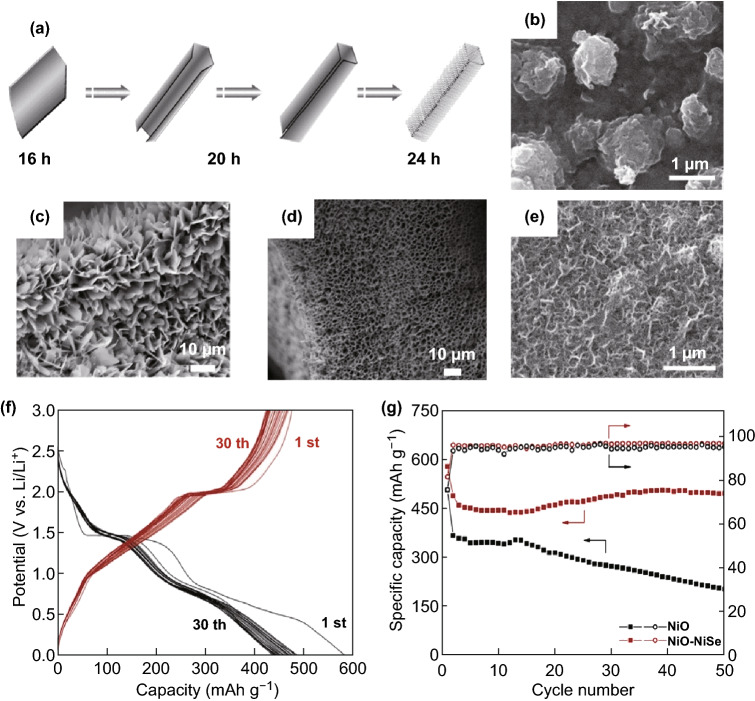
**a** Schematic diagram of the morphology evolution of nickel grown in situ on foamed nickel. **b** SEM image of NiO–NiSe electrode after initially discharging to 0.01 V. **c** Schematic diagram of the morphology evolution of nickel crystal grown in situ on foamed nickel with a reaction time of 16 h. **d** Schematic diagram of the morphology evolution of nickel crystal grown in situ on foamed nickel with a reaction time of 20 h. **e** SEM image of NiO–NiSe electrode after initially charging to 3.0 V. **f** At a current density of 20 mA g^−1^, the initial discharge and charge and discharge curves of the NiO–NiSe electrode in the voltage range of 0.1–3.0 V were 30. **g** Cycling performance of NiO–NiSe thin film electrode for the initial 50 cycles. Adapted from Refs. [46, 47] with permission. Copyrights: 2012, Royal Society of Chemistry and 2015, Elsevier

The discharge electrode shown in Fig. 4b has a rougher surface, where the micronized clusters are composed of nanosized particles. After the battery was recharged to 3.0 V, the micronized clusters of the material disappeared, and the electrode surface exhibited a honeycomb structure, as shown in Fig. 4e. The composite electrode had an initial discharge capacity of 577.7 mAh g^−1^ (Fig. 4f) and was able to maintain a capacitance of 495 mAh g^−1^ after 1 cycle and 50 cycles (Fig. 4g). The above investigation showed that the composite material also had good multiplier performance and discharge function. Even at the 2C rate, the capacity remains stable, which proves that the NiO–NiSe nanocomposite material can be decomposed reversibly and reformed during emission/charging. This study indicates that the NiO–NiSe composite will be an ideal choice for LIB anode materials in the future. In addition, Mi et al. [47] successfully designed tubular NiSe with a three-dimensional (3D) layered nanometer/microstructure via an *in situ* growth method (Fig. 4a). For the first time, the structure and shape of NiSe were adjusted by adjusting the reaction time. It was observed that by heat-treating the NiSe microplate, it could be entangled into microtubes over time (Fig. 4c, d). As an intermediate product, these microtubules could eventually be developed into hierarchical microtubules. In addition, Mi et al. determined that when the reaction temperature reached 180 °C, the solvent was converted into 10 mL of ethylenediamine and 6 mL of deionized water and then obtained another NiSe microtube composed of nanoparticles. The two types of NiSe microtubes obtained through this method can be used directly as working electrodes of a coin battery. A LAND system (model CT2001A) was used for charge and discharge cycles in the 1.0–3.0 V potential range. The charge and discharge reaction mechanisms of NiSe are as follows:

7 $${\text{NiSe}} + 2{\text{e}}^{ - }\, { \leftrightarrows }\,{\text{Ni}} + {\text{Se}}^{2 - }$$

8 $${\text{Li}} + {\text{Se}}^{2 - } \,{ \leftrightarrows }\,{\text{Li}}_{2} {\text{Se}} + 2{\text{e}}^{ - }$$

The initial reversible capacity was 410.7 mAh g^−1^, which was much larger than the theoretical capacity of 398.5 mAh g^−1^. However, Zhang et al. [48] developed a core–shell NiSe/C composite polymerization process using a two-step process. After five cycles, the core–shell NiSe/C composite had a reversible lithium storage capacity of 431 mAh g^−1^ and maintained a storage capacity of 428 mAh g^−1^ after 50 cycles.

#### Iron Selenium

In the past decade, porous and hollow nanomaterials with various morphologies and compositions have been extensively developed for energy storage, catalysis, gas sensors, drug delivery, and hydrogen precipitation reactions [49]. Furthermore, Liu et al. [50] successfully synthesized Fe_2_SeS through a simple solid inverse reaction and electrochemical research and reported, for the first time, the use of Fe_2_SeS as the anode of a LIB. The electrochemical reaction mechanism of Fe_2_SeS with lithium can be expressed as follows:

9 $${\text{Fe}}_{2} {\text{SeS}} + 4{\text{Li}}^{ + } + 2{\text{e}}^{ - } \,{ \leftrightarrows }\,{\text{Li}}_{2} {\text{Se}} + {\text{Li}}_{2} {\text{S}} + {\text{Fe}}$$

LIB electrochemical tests show that Fe_2_SeS can provide a large initial discharge capacity of 471 mAh g^−1^. Its initial coulombic efficiency (92.56%) was higher than those of most previously reported anionic compounds. The electrochemical reaction of Fe_2_SeS and Li has good reversibility. The Fe_2_SeS electrode has a high capacity and low discharge and charging levels and is thus a promising anode candidate for LIBs.

**Fig. 5:**
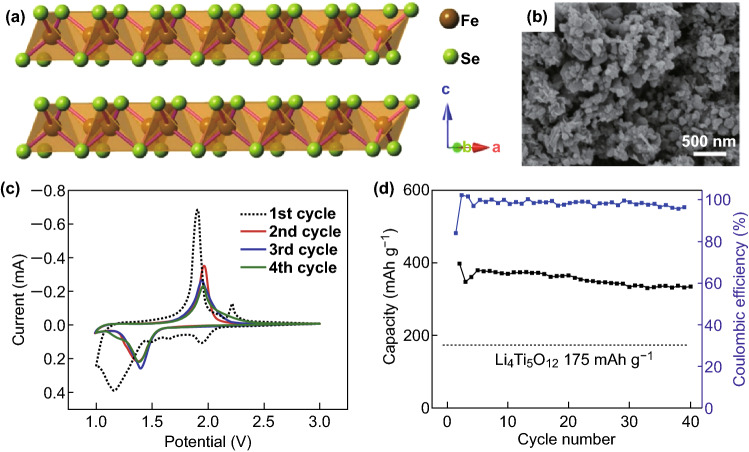
**a** Schematic of α-FeSe reveals the crystal structure of the layered structure. **b** SEM image of the sample. **c** The first four consecutive CVs of the α-FeSe@C composite have a scan rate of 0.1 mV s^−1^. **d** Cycle performance of the composite at a current density of 40 mA g^−1^. Adapted from Ref. [51] with permission. Copyright 2014, Elsevier

Wei et al. [51] synthesized a carbon-encapsulated α-FeSe (αFeSe@C) electrode material by a one-step reaction method (Fig. 5a). The surface-structured αFeSe@C composite was observed by SEM. The microscopic morphology is shown in Fig. 5b. The first four consecutive CV electrochemical properties of the product were evaluated at a scan rate of 0.1 mV s^−1^, as shown in Fig. 5c. The performance of the composite at a current density of 40 mA g^−1^ is shown in Fig. 5d. The potential energy storage and conversion applications can be observed from the performance chart analysis.

### Sodium-Ion Battery

Owing to its physical and chemical properties that are similar to the LIB, the SIB is considered an ideal energy storage technology [52]. The main challenge in SIBs is to find suitable electrodes, especially materials with excellent sodium storage properties. Owing to their physical and chemical properties bearing similarities, the materials used sometimes allow Li to embed the same crystal structure that is suitable for Na embedding. Therefore, all types of SIB electrode materials have the characteristics of LIB electrode materials [53]. In turn, the new electrode materials invented for use in LIBs are also of significance for the study of SIB electrodes. However, this strategy is not always feasible. Sodium resources are not only abundant and widely distributed in the earth’s crust but also cost less. Therefore, recently, room-temperature SIBs have received much attention from researchers, especially for large-scale power storage applications. The use of low-cost materials for electrodes is an important advantage of the SIB; however, the SIB poses enormous challenges compared to the LIB. The challenges faced with SIBs can be overcome by continuing research and constantly searching for new low-cost materials with good performance and stability [36].

#### Cobalt Selenium

**Fig. 6:**
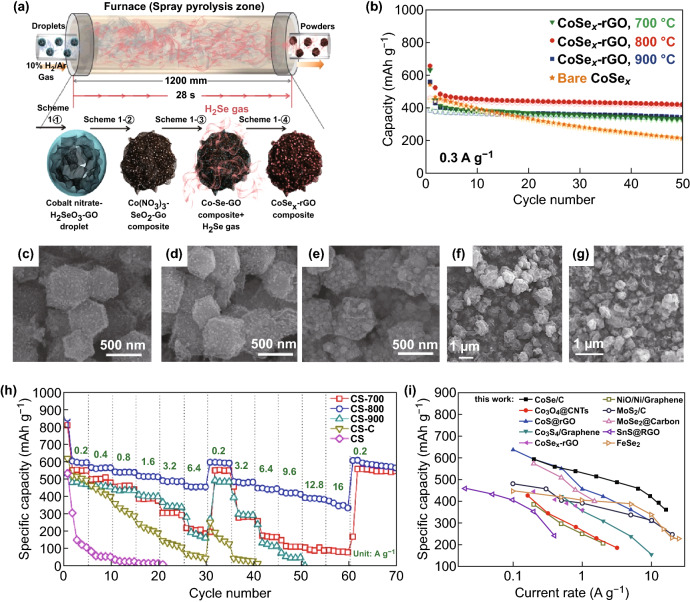
**a** Formation mechanism of CoSe_*x*_–rGO composite powder by one-pot spray pyrolysis process. **b** Electrochemical properties of the bare CoSe_*x*_ and CoSe_*x*_–rGO composite powders’ cycling performance. SEM and TEM images of the CoSe/C composites synthesized at different temperatures, **c** CS-700, **d** CS-800, **e** CS-900. SEM image, SAED pattern, and elemental mapping image of the CoSe_*x*_–rGO composite powders prepared at **f** 800 °C, **g** 900 °C. **h** Rate capability of the nitrogen-doped CoSe/C, CoSe/C, and bare CoSe. **i** Rate performances of the CS-800 electrode and many other anode materials for SIBs. Adapted from Refs. [54, 55] with permission. Copyrights: 2016, Wiley-VCH and 2017, American Chemical Society

Using a cobalt organic skeleton (ZIF-67) as the sacrificial template, Zhang et al. [54] studied the CoSe/C mesoporous dodecahedron formed by ZIF-67 and the Se powder at different temperatures in the original position of the nitrogen-doped carbon matrix. Figure 6c, d, e shows microscopic topographical views of dodecahedral CoSe/C composites formed at different temperatures. Because the CoSe nanoparticles are only approximately 15 nm in size, they can be trapped in the mesoporous carbon skeleton. As a new type of anode material for SIBs, the CoSe/C composite material exhibits high capacity and performance. When the current densities are 0.2 and 16 A g^−1^, the specific capacities of the composite electrodes are 597.2 and 361.9 mAh g^−1^, respectively. As an electrode material for SIBs, the CoSe/C dodecahedron has outstanding magnification, and its cycle stability is better (Fig. 6h). The transition-metal S prepared using this method also has high electrochemical properties. Park et al. [55] synthesized the CoSe_*x*_–rGO composite powder for a SIB anode material by the one-step synthesis. The specific synthesis process is shown in Fig. 6a. The microscopic topography of the CoSe_*x*_–rGO composite powder prepared under different conditions is shown in Fig. 6f, g. The results show that the CoSe_*x*_–rGO composite powder has better cycle performance than the bare CoSe_*x*_ powder, regardless of the preparation temperature. A composite powder mainly composed of the Co_0.85_Se and CoSe_2_ phases was prepared under spray pyrolysis conditions at 800 °C. At a constant current density of 0.3 A g^−1^, the discharge capacity of the two materials could be compared for the 50th and 2nd cycles, and the composite powder of CoSe_*x*_–rGO had a high density during repeated sodium-ion charging and discharging (Fig. 6b). Furthermore, the CoSe_*x*_–rGO composite powder has structural stability. Therefore, it has excellent sodium storage properties compared to the bare amphoteric powder. Later, Li et al. [41] used ZIF-67 as a precursor to embed advanced CoSe nanoparticles in a novel porous carbon polymer to produce a new CoSe@PCP composite for LIBs and SIBs. For the SIB, after 100 cycles of 100 mA g^−1^, its reversible capacity is 341 mAh g^−1^, with high cycle stability and excellent rate performance. Around the same time, Yang et al. [42] prepared a mixed CoSe_2_N-CF/CNT superstructure composed of internal CoSe_2_ nanoparticles and external CNT-entangled N-doped carbon skeletons through ZIF-67 by a simple two-step heat treatment procedure for LIBs and SIBs. The electrochemical reaction of CoSe_2_@N-CF/CNTs for sodium storage can be summarized as follows:

10 $${\text{Discharge}}\,{\text{process}}\;{\text{CoSe}}_{2} + x{\text{Na}}^{ + } + x{\text{e}}^{ - } \to {\text{Na}}_{x} {\text{CoSe}}_{2}$$

11 $${\text{Na}}x{\text{CoSe}}_{2} + \left( {2 - x} \right){\text{Na}}^{ + } + \left( {2 - x} \right){\text{e}}^{ - } \to {\text{CoSe}} + {\text{Na}}_{2} {\text{Se}}$$

12 $${\text{CoSe}} + 2{\text{Na}}^{ + } + 2{\text{e}}^{ - } \to {\text{Co}} + {\text{Na}}_{2} {\text{Se}}$$

13 $${\text{Charge}}\,{\text{process}}\;{\text{Co}} + 2{\text{Na}}_{2} {\text{Se}} \to {\text{CoSe}}_{2} + 4{\text{Na}}^{ + } + 4{\text{e}}^{ - }$$

Gao et al. [43] evaluated the potential application of CoSe_2_ MoSe_2_/C HNT as a candidate material for SIB anodes. Studies have shown that it has good storage efficiency and high stability for sodium.

#### Nickel Selenium

**Fig. 7:**
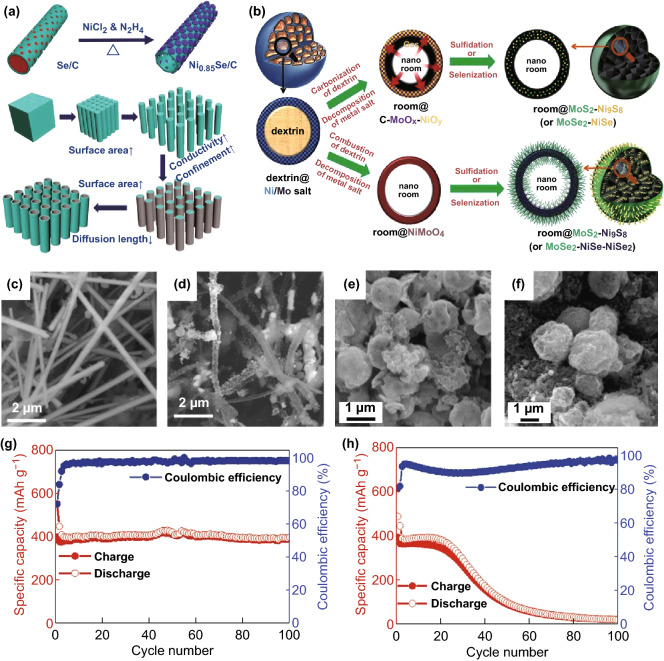
**a** Self-template method schematic and advantages of carbon-supported hollow nanowires on bulk materials. **b** Formation mechanism of hollow nanowires in MoS_2_–Ni_9_S_8_–C, MoSe_2_–NiSe–C, MoS_2_–Ni_9_S_8_, and MoSe_2_–NiSe_2_ microspheres. SEM images of **c** Se/C and **d** Ni_0.85_Se/C hollow nanowires at different magnifications. **e** Morphologies of the multiroom-structured MoX_2_-NiX_*y*_ (X_¼_ S or Se) after 100 cycles for MoSe_2_–NiSe–NiSe_2_ microspheres. **f** Morphologies of the multiroom-structured MoX_2_–NiX_*y*_ (X_¼_ S or Se) after 100 cycles for MoSe_2_–NiSe–C microspheres. **g** Specific capacity and coulombic efficiency of Ni_0.85_Se/C hollow nanowires. **h** Special ability and coulombic efficiency of Ni_0.85_Se nanoparticles at 0.2 C. Adapted from Refs. [56, 57] with permission. Copyrights: 2017, Wiley-VCH and 2017, The Royal Society of Chemistry

Yang et al. [56] used carbon-coated selenium nanowires as templates to synthesize carbon-loaded selenium–nickel hollow nanowires (Ni_0.85_Se/C). The specific synthesis method is shown in Fig. 7a. When used as a negative electrode material for SIBs, the Ni0_.85_Se/C nanowires provide a reversible capacity of approximately 390 mAh g^−1^ at a rate of 0.2 C (theoretical capacity of 416 mAh g^−1^) with no significant attenuation after cycling (Fig. 7g). However, in the first cycle, the irregular Ni_0.85_Se nanoparticles have a capacity of 390 mAh g^−1^ (Fig. 7h), which decreases to lower than 100 mAh g^−1^ after 50 cycles. Hollow nanowires provide excellent performance even under high-current conditions. Park et al. [57] used a pilot spray-drying process to prepare unique multicomponent metal S and Se microspheres. The specific synthesis of amylin decomposition in MoS_2_–Ni_9_S_8_ and MoSe_2_–NiSe_*x*_ microspheres produces empty nanoparticles, as shown in Fig. 7b. The microscopic morphology of the sample is shown in Fig. 7f, and the microscopic topography of MoSe_2_–NiSe–NiSe_2_ is shown in Fig. 7e. When the current density of sodium-ion storage was 0.5 A g^−1^, the capacity reserves measured in the second cycle were 91%, 102%, 92%, and 64%. The carbon-free MoS_2_–Ni_9_S_8_ microspheres exhibited excellent rate performance. When the current density increased from 0.1 to 3 A g^−1^, the discharge capacity decreased slightly from 559 to 428 mAh g^−1^. The MoS_2_–Ni_9_S_8_–C composite microspheres have high structural stability during multiple sodium-ion implantation and desorption processes and have high long-term cycle performance over 1000 cycles. In addition, Zhang et al. [48] synthesized a core–shell NiSe/C composite with a high sodium storage capacity (reversible lithium storage capacity of 339 mAh g^−1^ after 5 cycles and 280 mA h g^−1^ storage capacity after 50 cycles).

#### Iron Selenium

**Fig. 8:**
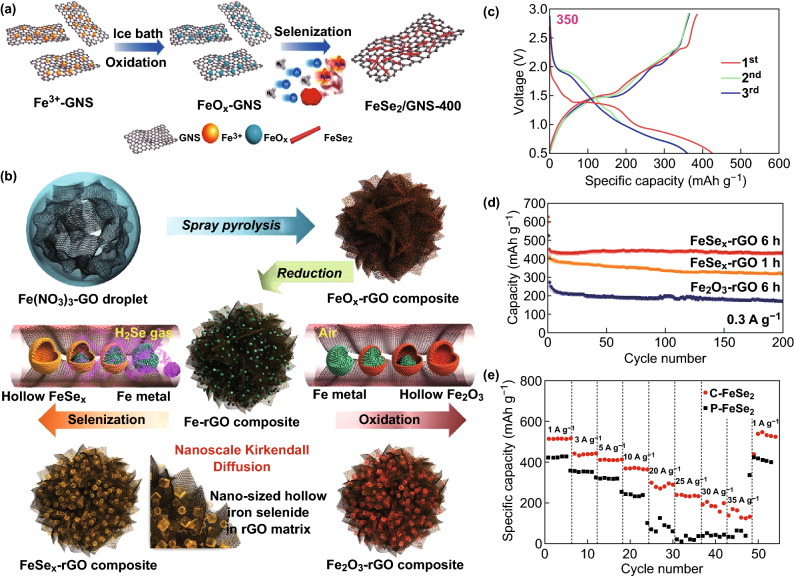
**a** Schematic diagram of the synthesis of FeSe_2_/GNS-400. **b** Hollow FeSe_*x*_ nanoparticles and hollow Fe_2_O_3_ nanoparticles modify the formation mechanism of rGO composite powder by nano-Gildo diffusion. **c** Initial three charge and discharge curves of FeSe_2_/GNS-300 and FeSe_2_/GNS-400. **d** Electrochemical performance of the hollow FeSe_*x*_- and Fe_2_O_3_-decorated rGO composite powders: cycling performances. **e** Electrochemical properties of the FeSe_2_ electrode prepared for SIB. Rate performance of FeSe_2_ clusters and particles after 50 cycles of current density from 1 to 35 A g^−1^. Adapted from Refs. [58–60] with permission. Copyrights: 2018, American Chemical Society; 2016, Nature and 2017, Tsinghua University Press and Springer-Verlag GmbH Germany

In designing a superior transition-metal selenide (TMS), the sodium-ion storage rate and cycle performance remain as significant challenges. Li et al. [58] uniformly immobilized FeSe_2_ on graphene nanosheets by the selenization of iron oxide. The specific synthesis process is shown in Fig. 8a. The surface oxide layer of FeSe_2_/GNS generates phase shift resistance at the bottom, inhibiting the formation of sodium ions and resulting in extremely poor storage kinetics of nanoions. It is worth noting that the surface of the prepared FeSe_2_ nanorod/graphene composite has almost no oxide layer and can reach a high capacity of 459 mA h g^−1^ (Fig. 8c). This strategy of controlling the surface oxidation to achieve high sodium-ion storage anodes has significant potential for other TMS designs. Later, Wei et al. [59] successfully synthesized a 3D FeSe_2_ cluster composed of nanorods using a simple hydrothermal method. As a SIB anode material, the prepared FeSe_2_ clusters have an excellent cyclic performance and a rate capacity up to 35 A g^−1^ (Fig. 8e). The unique 3D structure reduced the ion diffusion length and slowed down the slow kinetics. After 400 cycles at 1 A g^−1^, they had an initial coulomb efficiency of 97.4% and a discharge capacity of 515 mA g^−1^. Furthermore, we demonstrated that unique 3D structures, high capacitance, and appropriate etheric electrolytes contribute to the improved electrochemical performance. The excellent overall performance of the FeSe_2_ cluster makes it a promising anode material for SIBs. Finally, the FeSe_*x*_ reduced graphene oxide (RGO) composite powder prepared by Park et al. [60] using the nanoscale Kirkendall diffusion method has a unique structure. The FeSe_*x*_–rGO composite powder has a mixed-crystal structure of FeSe and FeSe_2_ phases (Fig. 8b). At a constant current density of 0.3 A g^−1^, the discharge capacities of the FeSe_*x*_– and Fe_2_O_3_–rGO composite powders were 434 and 174 mAh g^−1^, respectively (Fig. 8d). At a high current density of 1 A g^−1^, the discharge capacity of the FeSe_*x*_–rGO composite powder at 1600 cycles was 311 mAh g^−1^. The FeSe_*x*_–rGO composite powder has excellent sodium-ion storage performance compared with that of the Fe_2_O_3_–rGO composite powder with similar morphological characteristics. Xu et al. [61] designed a simple MOF-derived selenization strategy for the synthesis of in situ carbon-encapsulated selenides. When used as an excellent anode for a SIB, the uniform pod-like Fe_7_Se_8_@C nanorods had a current density of 3 A g^−1^ with a high specific capacity of 218 mAh g^−1^ after 500 cycles, and the porous NiSe@C sphere showed a high ratio of 160 mAh g^−1^ after the second cycle at a current density of 3 A g^−1^ capacity. Owing to the high energy capacity, transition-metal chalcogens have become the focus of researchers as a promising electrode material for SIBs. However, a limited cycle life and poor rate capabilities hamper their practical application. For the first time, Ali et al. [62] used a simple hydrothermal method to design and synthesize a graded porous iron–cobalt binary-metal selective (Fe_2_CoSe_4_, called FCSe) sphere using a unique selenization and annealing strategy. The synthesized FCSe was 816.3 mAh g^−1^ at 0.5 A g^−1^ and 400.2 mAh g^−1^ at 32 A g^−1^. The comprehensive strategy developed in this study opens a new approach. To achieve a high rate and long-term cycling of SIBs, a transition-metal-based binary-metal selenide can be produced.

### Other Batteries

#### Solar Cell

High power conversion efficiency (PCE) and cost-effectiveness are two long-term goals for dye-sensitized solar cells (DSSCs). Recently, Duan et al. [63] prepared a transparent, cost-effective Co–Se binary alloy without any surfactant or template, as a counterelectrode (CE) material in a DSSC through a mild solution strategy. The peak-to-peak separation (*E*_pp_) and *R*_ct_ of the Co_0.85_Se alloy CE were lower than those of other electrodes; however, the CE exhibited a higher peak current density. It is worth noting that the DSSC front-and-back efficiencies of the Co_0.85_Se alloy CE were 8.30% and 4.63%, respectively, which indicated superior battery performance to that of the original Pt electrode. The advantages of such a double-sided solar cell system with a fast start-up, high multistart capability, low electronic recombination, and high photocurrent stability make this solar cell system potentially useful in vehicles, engines, and power supplies. The test results of the double-sided radiation technology show that it paves the way for cost-effective, efficient, and practical DSSCs. Owing to their cost-effectiveness, high electrical conductivity, good electrocatalytic activity, and reasonable stability, alloy materials have become an alternative electrocatalyst for electrochemical devices. To reduce the manufacturing costs without sacrificing the DSSC PCE, Liu et al. [64] reported the feasibility of designing a transparent and cost-effective Fe–Se alloy CE for the double-sided DSSC. Owing to the rapid charge transfer capability and electrocatalytic activity, the maximum pre- and post-efficiency values of the DSSC using the Fe alloy electrodes were 7.64% and 4.95%, respectively, whereas these values for the Pt-based solar cells were 6.97% and 3.56%, respectively. The impressive results and simple synthesis highlight the potential applications of Fe–Se alloys in robust double-sided DSSCs.

#### Aqueous Battery

Nickel, cobalt oxide, sulfur, and selenium have been extensively studied by researchers as active substances. As the number of atoms of nonmetallic elements increases, enhanced capacities in energy storage applications are obtained. Li et al. [65] synthesized NiCoO_2_ by the hydrothermal method. They then prepared their corresponding sulfides and selenides by a corresponding ion exchange and studied the electrochemical process as the electrode material of a water battery. The results show that the local environment created by the electronic structures of S and Se leads to more Ni and Co cation activities. However, selenide exhibits a severely degraded performance during cycling, and S and Se losses are detected after the CV test, which causes a serious decrease in the capacity of sulfide and selenide. This study has some significance for the use of nickel, cobalt, sulfur, or selenium nanostructures for aqueous batteries in the future.

#### Others

Some researchers have studied the use of transition-metal selenides in lithium–sulfur batteries. Xue et al. [66] successfully produced nickel diselenide by reactive pulsed laser deposition and checked the electrochemical behavior of NiSe_2_/Li cells by a constant current cycle measurement and CV to achieve a large reversible discharge capacity of 351.4 mAh g^−1^ for the NiSe_2_/Li battery. According to the results of in situ XRD, TEM, and SAED, the electrochemical reaction mechanism of NiSe_2_ and lithium was proposed as follows:

14 $${\text{Discharge}}\,{\text{process}}\,\,{\text{NiSe}}_{2} + 2{\text{Li}} \to \beta - {\text{NiSe}} + {\text{Li}}_{2} {\text{Se}}\,\left( {2.43{-}1.65\,{\text{V}}} \right)$$

15 $$3\beta - {\text{NiSe}} + 2{\text{Li}} \to {\text{Ni}}_{3} {\text{Se}}_{2} + {\text{Li}}_{2} {\text{Se}}\,\left( {1.65{-}1.5\,{\text{V}}} \right)$$

16 $${\text{Ni}}_{3} {\text{Se}}_{2} + 4{\text{Li}} \to 3{\text{Ni}} + 3{\text{Li}}_{2} {\text{Se}}\,\left( {1.5{-}1.0\,{\text{V}}} \right)$$

17 $${\text{Charge}}\,{\text{process}}\,\,3{\text{Ni}} + 2{\text{Li}}_{2} {\text{Se}} \to {\text{Ni}}_{3} {\text{Se}}_{2} + 4{\text{Li}}\,\left( {1.0{-}2.15\,{\text{V}}} \right)$$

18 $${\text{Ni}}_{3} {\text{Se}}_{2} + {\text{Li}}_{2} {\text{Se}} \to 3\beta - {\text{NiSe}} + 2{\text{Li}}\,\left( {2.15{-}2.4\,{\text{V}}} \right)$$

19 $$\beta - {\text{NiSe}} + {\text{Li}}_{2} {\text{Se}} \to {\text{NiSe}}_{2} + 2{\text{Li}}\,\left( {2.4{-}3.0\,{\text{V}}} \right)$$

In this study, Yang et al. [67] designed a CoSe_2_–porous carbon composite (CoSe_2_–PC), which was prepared by a simple annealing process, as a high-performance cathode material for LIBs. Se@CoSe_2_–PC has a reversible capacity of up to 408 mAh g^−1^ after 100 charge–discharge cycles at a current rate of 1C, and its performance is much better than that of porous carbon alone. Symmetrical battery detection of Se@CoSe_2_–PC in the presence and absence of polyselenide in the dielectric indicates that the Li–Se battery based on Se–CoSe_2_–PC has an excellent cycle performance. This enables the inhibition of the dissolution of polyselenide to ultimately achieve the high capacity and cycle stability of Li–Se batteries.

### Summary

From the aforementioned research on electrochemical energy storage devices, it can be observed that the LIB and SIB have been widely studied as highly efficient electrochemical energy storage devices. By comparing the data in Table 1, it is clear that LIBs have higher specific capacities and longer cycle lives than SIBs. Nevertheless, SIBs are widely used because of their rich sodium-ion content and low redox potential. Researchers have also studied other types of batteries, such as solar cells and water-based batteries. The type, structure, and size of composite materials influence the electrochemical energy storage of various types of batteries. Comparing the literature, it is found that porous, core–shell, honeycomb, and 3D layered nanostructure tubular structures can effectively increase the ion transport space and shorten the ion and electron transport pathways, thereby improving the cycle stability of a battery [68].

**Table 1:** Application of iron/cobalt/nickel selenides in LIBs and SIBs

Sample	Method	DC^a^ (mAh g^−1^)	b-DC^b^ (mAh g^−1^)	Cycles	CD^c^ (mA g^−1^)	References
*LIBs*						
CoSe_2_@CNFs	Electrospinning	845	941	100	200	[39]
CoSe@carbon nanoboxes	Annealed	796	860	100	0.2	[40]
NiO–NiSe	Pulsed laser deposition	577.7	495	50	20	[46]
NiSe	In situ growth	410.7	–	4	4.2	[69]
Fe_2_SeS	Solid inverse reaction	471	397.2	100	0.1C	[50]
NiSe_2_	Pulsed laser deposition	467.5	318.7	100	5	[66]
FeSe@C	One-pot	390	340	40	40	[70]
*SIBs*						
CoSe/C	Annealing	–	531.6	50	5000	[54]
CoSe_*x*_–rGO	One-pot synthetic	656	400	50	300	[55]
Ni_0.85_ Se/C	Hydrothermal method	397	–	100	0.2 C	[56]
(Mo, Ni) metal selenide	Pilot-scale spray drying	546	–	80	500	[57]
Fe_7_Se_8_@C nanorods	Situ carbon-encapsulating	–	360	100	300	[61]
NiSe@C spheres	Situ carbon-encapsulating	–	325	45	300	[61]
Fe_2_CoSe_4_	Hydrothermal method	750	–	–	1000	[62]
FeSe_2_/GNS	Pulsed laser deposition	459	–	–	100	[58]
FeSe_*x*_–rGO	Nanoscale Kirkendall diffusion method mechanism	–	434	200	1000	[71]

All values were calculated based on the total weight of the whole electrode

^a^DC, initial specific capacity

^b^DC, final specific capacity

^c^D, current density

## Supercapacitor

As a potential new type of material, SCs concentrate large amounts of energy in the form of electric charges and can release large amounts of energy in a short time. SCs have the advantages of working quickly, long life, high rate density, short charge–discharge time, and so on. However, their electrode material is a key link that directly determines the performance, output rate, and efficiency of the entire SC device [72]. However, the application of SCs as important energy storage devices is limited owing to their low energy density. This paper introduces a method to improve their energy density by improving the electrode material.

### Cobalt Selenium

Compared with other chalcogenides (such as O and S), Se has a lower electronegativity and larger ionic radius. For cobalt, the outermost orbital electrons are less attractive to selenide; instead, weakly bound electrons are prone to redox reactions. However, the provision of electroactive sites can improve the overall kinetics of the electrochemical reaction. The semiconducting properties of cobalt selenide and its low optical band gap have led to its wide use in the fields of catalysis, DSSC, and electrochemical energy storage systems in recent years [33, 34]. Zhu et al. [73] applied a 3D interconnected ultrathin CoSe nanosheet synthesized by the hydrothermal method to the field of SCs for the first time. Experimental studies have shown that 3D interconnected ultrathin CoSe nanosheets have a high specific capacity and significant multiplication performance. The 3D interconnected ultrathin CoSe nanosheets and activated carbon (AC) were assembled as the cathode and anode of a novel aqueous hybrid SC. The assembled capacitor mixing unit exhibited a specific energy of 18.6 Wh kg^−1^ at a specific power of 750 W kg^−1^. The aqueous hybrid SC had excellent coulombic efficiency (~ 100%) and excellent cycle life during charge–discharge cycles. According to the above research results, the new 3D interconnect ultrathin CoSe nanosheet material can be used as a potential electrode material for hybrid SCs owing to its good electrochemical performance. The redox peak can be attributed to the Faraday redox reaction between Co^2+^/Co^3+^ and Co^3+^/Co^4+^, and its redox principle can be expressed as follows:

20 $${\text{CoSe}} + {\text{OH}}^{ - } \,{ \leftrightarrows }\,{\text{CoSeOH}} + {\text{e}}^{ - }$$

21 $${\text{CoSeOH}} + {\text{OH}}^{ - }\, { \leftrightarrows }\,{\text{CoSeO}} + {\text{H}}_{2} {\text{O}} + {\text{e}}^{ - }$$

To improve the electrochemical performance of SCs, Ma et al. [74] synthesized sheet-like Bi_18_SeO_29_/BiSe and flower-like Co_0.85_Se nanosheets as the negative and positive electrodes of the full tantalum capacitor using a low-temperature one-step hydrothermal method. The electrochemical performance test of the SC device assembled with Bi_18_SeO_29_/BiSe and Co_0.85_Se shows that it has a high specific capacitance (471.3 and 255 F g^−1^ at 0.5 A g^−1^), excellent cycle stability, and high conductivity. The prepared tantalum capacitor has good cycle stability (a capacity retention rate of 93% after 5000 cycles at a current density of 2 A g^−1^). The research results show that the above preparation method is simple and easy, and it is a promising method for asymmetric SCs (ASCs). Balakrishnan et al. [75] synthesized a cobalt selenide–graphene (CoSe–G) nanocomposite and pure CoSe using a simple one-step hydrothermal method. The reaction mechanism of CoSe in an alkaline solution is as follows:

22 $${\text{CoSe}} + {\text{OH}}^{ - } \,{ \leftrightarrows }\,{\text{CoSeOH}} + {\text{e}}^{ - }$$

**Fig. 9:**
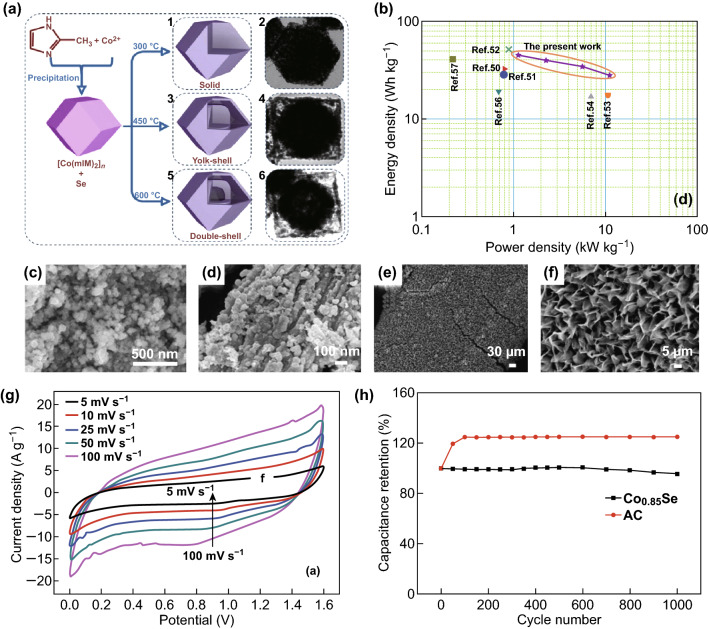
**a** Solid preparation process diagram. **b** Ragone plots of an ASC device (energy density vs. power density). **c** FE-SEM image of CoSe nanoparticles. **d** FE-SEM image of a CoSe-G nanohybrid. **e, f** FE-SEM image of Co_0.85_Se nanosheets on Ni foam. **g** CV curve range of ASC at different scanning rates: 5–100 mv s^−1^. **h** Electrochemical performance of Co_0.85_Se nanosheets, electrode cycling is 5 A g^−1^. Adapted from Refs. [75–77] with permission. Copyrights: 2016 (2017), Elsevier and 2017, The Royal Society of Chemistry

The microstructure of CoSe–G is shown in Fig. 9c, d. As shown in Fig. 9g, the assembled ASC can achieve a wide operating voltage of 1.6 V. The ASC has high power and energy densities and can maintain its initial specific capacity of 81.7% even after 5000 charge–discharge cycles (Fig. 9b). The test results show that the synergy between graphene and CoSe, together with the contribution of the two-layer material and the Faraday material, improves the overall electrochemical performance of the CoSe–G nanocomposite.

Zhang et al. [76] used MOFs as a sacrificial template to prepare the CoSe_2_/C dodecahedron with an adjustable internal structure. The specific synthesis process is shown in Fig. 9a. As an electrode material for SCs, CoSe_2_/C composites have the advantages of a high specific capacity, high rate performance, and long-term stability. Gong et al. [77] grew mesoporous Co_0.85_Se nanosheets on foamed nickel as an SC electrode material by a simple hydrothermal method. The microscopic morphology of the Co_0.85_Se nanosheet coated on the Ni foam is shown in Fig. 9e, f. As shown in Fig. 9h, when the current density is 5 A g^−1^, the Co_0.85_Se nanosheets electrode material has a specific capacitance of 1378 F g^−1^ and still has 95.5% retention after 1000 cycles. Using AC as the anode material, the asymmetric configuration of the asymmetric SC prepared by using a Co_0.85_Se nanosheet as the cathode material can extend the voltage window to 1.6 V; moreover, the material exhibits higher power density, energy density, and coupling cycle stability. The results show that mesoporous Co_0.85_Se nanosheets are promising as SC materials.

### Nickel Selenium

Recently, researchers have begun to pay attention to materials such as transition-metal oxides and sulfides when studying SC materials. Similar to oxygen and sulfur, selenium is also located in the sixth main group of the periodic table, and thus, it has chemical and physical properties similar to those of oxygen and sulfur. Selenium exhibits better metal properties than sulfur so that transition-metal selenides can be used in SCs [40–42]. NiSe is a promising electrode material, because it has various titanium oxides and good charge transfer conductivity [43, 44]. Therefore, we can improve the electrochemical properties of the electrode materials of SCs by controlling their morphology and structure [78].

**Fig. 10:**
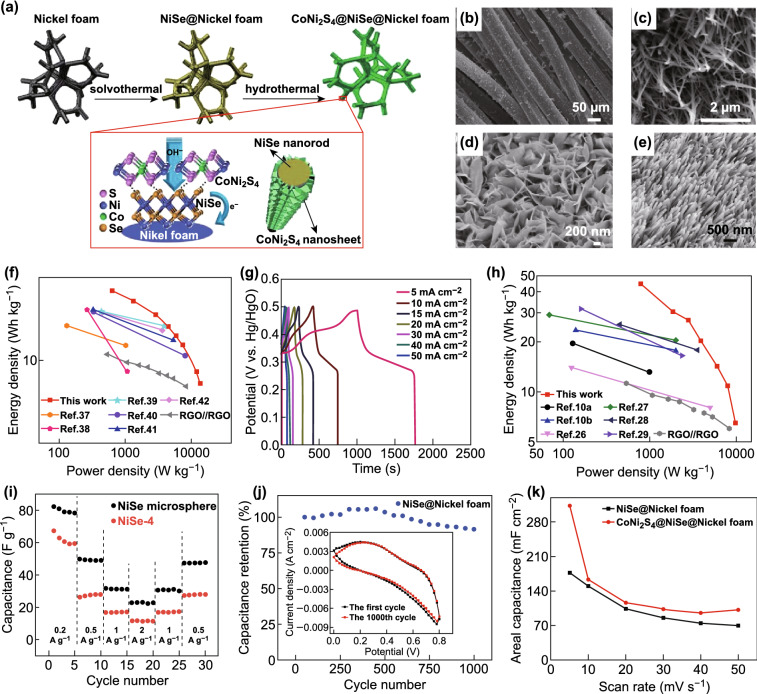
**a** Schematic representation of in situ growth of CoNi_2_S_4_@NiSe nanoarrays on compressed foamed nickel. **b** FE-SEM image of NiSe_2_/CFC. **c** SEM image of (Ni, Cu) Se_2_-0.30. **d** FE-SEM image of NiSe@MoSe_2_ nanosheet arrays. **e** SEM image of NiSe-0.20. **f** Ragone plot of SCs. **g** GCD curves for different doses of NISE-0.20 at different current densities. **h** Ragone plot of SCs. **i** Electrochemical performance of ASCs based on NiSe microspheres and NiSe microsphere discharge specific capacitance curves. **j** Flexible SC based on NiSe@nickel foam has a cycle performance of 1000 cycles at a scan rate of 50 mV s^−1^. **k** Areal capacitance dependence on scan rates of flexible SCs based on the NiSe@nickel and CoNi_2_S_4_@NiSe@nickel foam. Adapted from Refs. [80–84] with permission. Copyrights 2016 (2017), The Royal Society of Chemistry; 2017, Elsevier; and 2017, Wiley-VCH

Recently, Peng et al. [79] used a one-step hydrothermal method to synthesize nickel foam (NF) as a nickel precursor and a nucleation skeleton to synthesize a novel positive electrode material NiSe@ MoSe_2_ with a uniform vertical nanoarray structure and a high specific capacity. The microstructure of NiSe@MoSe_2_ is shown in Fig. 10d. We constructed a new NiSe@ MoSe_2_//N-PMCN ASC device using an N-PMCN electrode and a NiSe@MoSe_2_ nanosheet with excellent electrochemical properties and a unique spatial structure. The stability of the ASC device fabricated at the operating potential window of 1.65 V is very good, with a high energy density of 32.6 Wh kg^−1^ at a power density of 415 W kg^−1^. Furthermore, the capacity retention after 5000 cycles was 91.4%. This study shows that the establishment of stable heterostructures in promising electrode materials has much potential, and a new approach has been proposed to fabricate ASCs in low-cost biomass carbon materials. Capacitor assemblies are fabricated on the basis of high-performance Faraday electrode materials. Based on this research, Tian et al. [80] synthesized a NiSe nanorod array (NiSe NRA/NF) on an NF material by a one-step hydrothermal method. The microstructure is shown in Fig. 10e. When the current density is 5 mA cm^−2^, it has an ultrahigh specific capacitance of 6.81 F cm^−2^ (Fig. 10g). An ASC was assembled by using NiSe as a positive electrode and RGO as a negative electrode. The energy density was 38.8 Wh kg^−1^ at a power density of 629 W kg^−1^. The energy density was 6.38 Wh kg^−1^ at a power density of 13.5 kW kg^−1^ (Fig. 10f). Moreover, at a high current density of 3.6 A g^−1^, the device remained at 90.09% after 3000 cycles, thereby showing promise for applications. However, Chen et al. [81] used a simple one-step hydrothermal NF to prepare (Ni, Cu) Se_2_ nanowires for use as positive electrodes for ASCs. Their microscopic topography is shown in Fig. 10c. An (A, Cu) Se_2_ nanowire/NF was used as a positive electrode, and RGO was used as a negative electrode to assemble an ASC with an energy density of 6.53 Wh kg^−1^ at a power density of 9796.7 W kg^−1^ (Fig. 10h). After 4000 cycles of an ultrahigh current density of 50 mA cm^−2^, the energy density remained at 97.56%. As far as we know, this is the first (Ni, Cu)Se_2_ SC/ASC. For large-scale practical SCs, this work may motivate researchers to study transition-metal selenides and metal selenides. This design generates new interest. In addition, prior to the above studies, many researchers attempted different methods to synthesize nickel selenide-based compounds for use in SCs; for example, Bao et al. prepared a nickel selenide/carbon fiber cloth (NiSe_2_/CFC) flexible electrode by a simple and effective electrodeposition method at room temperature. As seen from Fig. 10b, large particles of NiSe_2_ uniformly cover carbon fibers. Owing to the high conductivity of NiSe_2_ nanostructures, the synthesized electrodes have excellent rate performance, high specific capacitance, and perfect tantalum capacitance. NiSe_2_/CFC electrodes have a higher rate performance in a three-electrode system. (The performance ratio varies between 1058 and 996.3 F g^−1^ when the current density varies between 2 and 10 A g^−1^.) In addition, the power density and energy density of the ASC composed of the AC electrode and the NiSe_2_/CFC electrode were 800 W kg^−1^ and 32.7 Wh kg^−1^, respectively. The ASC can still maintain 86% capacitance after 2000 cycles, with good durability and cycle stability. Therefore, the use of NiSe_2_/CFC as an electrode for a flexible energy storage device is very promising [82]. The reversible redox reaction mechanism of NiSe can be expressed as follows:

23 $${\text{NiSe}}_{2} + {\text{OH}}^{ - } \leftrightarrow {\text{NiSe}}_{2} {\text{OH}} + {\text{e}}^{ - }$$

24 $${\text{NiSe}}_{2} {\text{OH}} + {\text{OH}}^{ - } \leftrightarrow {\text{NiSe}}_{2} {\text{O}} + {\text{H}}_{2} {\text{O}} + {\text{e}}^{ - }$$

For the purpose of designing and manufacturing flexible SCs with high-performance electrode materials, Tang et al. [83] used a three-step hydrothermal method to synthesize a layered CoNi_2_S_4_@NiSe nanoarray in situ on compressed foamed nickel. The synthesis method is shown in Fig. 10a. A flexible SC based on a CoNi_2_S_4_@NiSe nanoarray had high surface capacitance (surface capacitance 312.95 mF cm^−2^ at a scan rate of 5 mV s^−1^) (Fig. 10k) and good cycle stability (1000 cycles). In addition, the capacitance retention rate was 97.59% (Fig. 10j). It also has excellent electrochemical Al stability and maintains good capacitance even under bending conditions. The layered structure of the CoNi_2_S_4_@NiSe nanoarray makes high-performance flexible SCs as one of the most promising electrode materials.

Guo et al. [84] prepared a layered nanosheet-based NiSe microsphere by a simple hydrothermal method. The nanochip-based NiSe microspheres exhibited excellent electrochemical properties when used as an electrode material for an SC (the specific capacitance at a current density of 0.5 A g^−1^ was 492 F g^−1^). In addition, an ASC with a specific capacitance of 80 F g^−1^ at a current density of 0.2 A g^−1^ was successfully fabricated using the previously prepared material (Fig. 10i). This work greatly enriches the knowledge on the types of SC electrode materials and provides effective guidance for the practical application of NiSe electrode materials. The redox mechanism of NiSe in an aqueous alkaline solution can be expressed as:

25 $${\text{NiSe}} + {\text{H}}_{2} {\text{O}} + 1/2{\text{O}}_{2} \to {\text{Ni}}\left( {\text{OH}} \right)_{2} + {\text{Se}}$$

26 $${\text{Ni}}\left( {\text{OH}} \right)_{2} + {\text{OH}}^{ - } \,{ \leftrightarrows }\,{\text{NiOOH}} + {\text{H}}_{2} {\text{O}} + {\text{e}}^{-}$$

### Bimetallic Selenium

Researchers have studied various metal complexes and found that when multiple metals are present, there may be mutual promotion. Yang et al. [85] developed a series of methods for in situ growth in a (Ni_*x*_Co_1−*x*_)_9_Se_8_ solid solution. When the current density is 5 A g^−1^, the (Ni_*x*_Co_1−*x*_)_9_Se_8_ nanocrystal has a specific capacitance of 3566 F g^−1^ after 5000 cycles. The assembled flexible (Ni_0.1_Co_0.9_)_9_Se_8_@CFC//PVA/KOH//rGO@CFC device with ultrahigh energy density was better than the recently reported tantalum capacitor based on nickel–cobalt S and selenide counterparts. These (Ni_*x*_Co_1−*x*_)_9_Se_8_ nanodendrites may also be efficient and robust electrocatalysts for hydrogen evolution reaction (HER) and bulk water splitting. Further research on the electrocatalytic performance is still in progress. Using the one-step hydrothermal method, Chen et al. [86] synthesized electrode materials with similar hollow structures but different NiSe and CoSe ratios. When such an electrode material was used for an energy storage active material with a positive electrode, it was found that the NiSe–CoSe sample exhibited excellent electrochemical activity in the alkaline electrolyte. Owing to the synergistic effect between NiSe and CoSe, we adjust the electrochemical performance by adjusting their ratio. The experimental results show that when the NiSe to CoSe ratio is 4:2, the NiSe–CoSe sample exhibits the best electrochemical performance as an electrode material. In addition, the hybrid SC has higher power and energy densities (the energy density is 41.8 Wh kg^−1^ at a power density of 750 W kg^−1^; at a power density of 30 kW kg^−1^, the energy density is 20.3 Wh kg^−1^). Zhang et al. [87] synthesized a Co_0.85_Se nanosheet array consisting of uniform (3–5)-nm ultrasmall Co_0.85_Se nanocrystals by one-step electrodeposition. In addition, it was also demonstrated that a 3D layered Co_0.85_Se@NiCo_2_S_4_ was designed and fabricated on graphene foam (GF) with a capacitance of 10 F cm^−2^ at a scan rate of 1 mV s^−1^. When the current density is zero, it has surface capacitance values of 5.25 and 2.65 F cm^−2^. In addition, the constructed flexible solid-state ASC device has good power and energy densities within the 1.55-V electrochemical potential window. The solid-state ASC device still has a capacitance retention rate of 89.0% after 10,000 cycles, and it can be observed that the device has excellent electrochemical stability. The successful construction of Co_0.85_Se on NiCo_2_S_4_ nanotube arrays on GF opens the possibility of selectively depositing transition metals on various 3D substrates.

### Summary

As a new type of electrochemical energy storage device, SCs have attracted considerable attention from researchers. The employed material is the key factor in determining the performance of the device. The discussion above elucidated studies on the application of iron–cobalt–nickel-based materials in SCs. The data in Table 2 show that the spatial structure of the sample has significant influence on the performance of the SC. Materials with a dual-core structure show better electrochemical performance than those with a mononuclear structure and a solid material. The spatial structure of the material determines the surface area and pore volume of the material to some extent. Therefore, it has a certain influence on the electron transfer and redox field, which in turn affect the electrochemical properties of the material. In addition, the consistency of the multimetal can also improve the electrochemical performance of the SC to some extent.

**Table 2:** Transition-metal sulfides based on graphene or graphene derivatives for SCs

Materials	SC^a^ (F g^−1^)	Cycles	CD (A g^−1^)	PD^d^ (W kg^−1^)	ED^c^ (Wh kg^−1^)	CR^b^ (%)	References
CoSe nanosheets	–	20,000	5	18,000.75	18.6	95.4	[73]
Co_0.85_Se	255	5000	2	–	–	93	[74]
CoSe–G	1016	5000	1	11.2	45.5	81.7	[75]
CoSe_2_/C	–	5000	10	–	–	84.7	[76]
Co_0.85_Se	1378	2000	1	789.6	39.7	95.5	[77]
NiSe@MoSe_2_	–	5000	5	85,922	415	91.4	[79]
NiSe NRA/NF	–	3000	0.05	629	38.8	90.9	[80]
CoNi_2_S_4_@NiSe	851.9	1000	–	–	30.97	89.73	[83]
NiSe microspheres	40	2000	0.5	–	–	100	[84]
(NixCo_1−*x*_)_9_Se_8_	3566	5000	5	–	–	94.8	[85]
NiSe-CoSe	426	6000	7	750	41.8	91.7	[86]
Co_0.85_Se@NiCo_2_S_4_ CoNi_2_S_4_/G	10	10,000	–	750	46.5	89.5	[87]

^a^*SC* specific capacitance

^b^*CR* capacitance retention

^c^*ED* energy density

^d^*PD* power density

## Electrocatalysis

### Oxygen Reduction Reaction

The ORR is an important part of electrochemical energy conversion and requires four electrons for a given catalyst. In recent years, it has been found that transition metals such as Mn [88], Fe [89, 90], Co [91, 92], Ni [93, 94], and Cu [95] and heteroatom dopants B [96], N [97, 98], P [99, 100], S [101, 102], and Se [103] can change the catalytic properties of various carbon materials, including CNTs, amorphous carbon, and graphene. This drove many researchers to study and develop nonprecious metal catalysts. Owing to their high selectivity to the ORR in acidic and basic media, transition metals have good electrocatalytic activity.

#### Cobalt Selenium

**Fig. 11:**
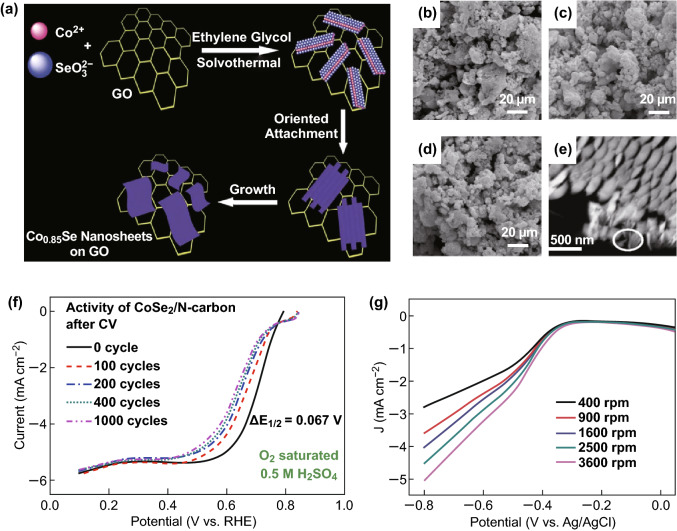
**a** Schematic diagram of the synthesis of ultrathin Co_0.85_Se/graphene nanocomposites. SEM micrograph of synthesized Co–Se catalyst powder, **b** 31.7 mol% Se, **c** 67.1 mol% Se. **d** 70.9 mol% Se. **e** In progressively enlarged magnified image, the slice sample of the component Co (44%) Se (56%). **f** Stabilities of CoSe_2_/N–carbon catalysts. The activity decay after 1000 CV cycles, DE_1/2_, is marked on the graph. **g** RDE curve of Co_0.85_Se/graphene (loaded approximately 0.1 mg cm^−2^) in O^2−^-saturated 0.1 M KOH was 10 mV s^−1^ at different speeds. Adapted from Refs. [104–106, 108] with permission. Copyrights: 2014, The Royal Society of Chemistry and 2012 (2006, 2013), Elsevier

Compared with the corresponding bulk samples, the advantages of ultrathin nanosheets are more prominent. Ultrathin nanosheets have relatively novel physical properties and electronic structures. However, they still have certain challenges owing to the inherent forces driving the anisotropic growth of 2D structures. Zhang et al. [104] used a simple thermal solvent method to synthesize a large ultrathin single-crystal Co_0.85_Se nanosheet with a thickness of 3 nm in situ on a graphitized graphene (GO) plate (Fig. 11a). The synergistic chemical coupling between GO and Co_0.85_Se in the Co_0.85_Se/GO nanocomposites showed the highest catalytic performance of the ORR cobalt chalcogenide-based catalysts (Fig. 11g). In addition, the Co_0.85_Se/GO hybrid nanoparticle can promote rapid decomposition of hydrazine hydrate (97% of the hydrazine hydrate degrades in 12 min), and its degradation rate can be kept constant for 10 consecutive cycles; therefore, Co_0.85_Se/GO nanocomposites have the potential to act as catalysts for a long time. Super et al. [105] combined CoSe_2_ with nitrogen-doped carbon in several catalysts. Among them, the CoSe_2_/N–C catalyst synthesized at 400 °C showed the highest activity. CoSe_2_/N–C has an active carrier N–Cm, which has high stability and short activity decay. In the 0.5-M H_2_SO_4_ electrolyte, the half-wave potential (*E*_1/2_) of the N-carbon decreased from 0.667 to 0.636 V after 1000 cycles, whereas the *E*_1/2_ value of the active substance CoSe_2_/N-C decreased from 0.711 to 0.644 V. In contrast, the *E*_1/2_ value of CoSe_2_/C decreased from 0.681 to 0.475 V (Fig. 11f). Some researchers have synthesized compounds related to cobalt selenide by heating and studied their ORR activity. Zhao et al. [106] were the first to heat elemental selenium and hexadecylcarbonyltetracobalt [Co_4_(CO)_12_] under reflux conditions in the 1,6-hexanediol solvent to synthesize Co-based selenide with various selenium contents. The experimental results show that cobalt-based selenium exhibits excellent electrochemical stability in the potential range of 0.05–0.80 V (vs. NHE). In Fig. 11b, c, and d, the microtopographies show that the sample exhibits a surface morphology similar to broccoli and shows the crystalline character of the orthogonal CoSe_2_ compound. The experimental results show that, in the electrolyte solution of 0.5 M H_2_SO_4_, the catalytic activity is the highest when the content of Se is 67.1 mol% and the OCP value is 0.79 V (NHE). A Tafel slope of − 60 mV dec^−1^ and a transfer coefficient of 0.49 were obtained in the kinetic control region. Therefore, the main factor affecting the performance of the co-selenium chalcogenide catalyst may be the modification of selenium on the metal structure of the metal cobalt. Subsequently, Nekooi et al. [107] microwaved sodium selenite and cobalt (II) acetate in a glycerol solution to synthesize a CoSe catalyst supported on nanoporous carbon. The current amplitude and the initial potential of the ORR have almost the same effect on 20 wt% CoSe/C in the presence and absence of these fuels. Because oxidation and oxygen reduction occur simultaneously, the Pt/C catalyst exhibits a mixed potential while causing the initial potential of the ORR to decrease at approximately 0 °C. The electrochemical measurements indicate that the synthesized CoSe/C catalyst has a four-electron transfer mechanism of ORR. The above results indicate that electrocatalysts, which have various fuel capacities, low cost, and nearly complete tolerance, are suitable for use in mixed and conventional reaction fuel cells fueled with low molecular weight alcohols or formic acid. However, Zhu et al. [108] prepared a Co–Se film by magnetron sputtering. The film showed remarkable activity for ORR. The film has been shown to contain Co–Se nanocrystals embedded in a Se-rich matrix (Fig. 11e). The structure of the Co–Se nanocrystals participating in the ORR is different from the structure based on the Chevrel phase. For the Co–Se catalyst, the questions of how the Co_1−*x*_Se nanocrystallites are related to the active site of electron transfer and how the surrounding Se enrichment contributes to the entire ORR process remain open.

#### Iron Selenium

**Fig. 12:**
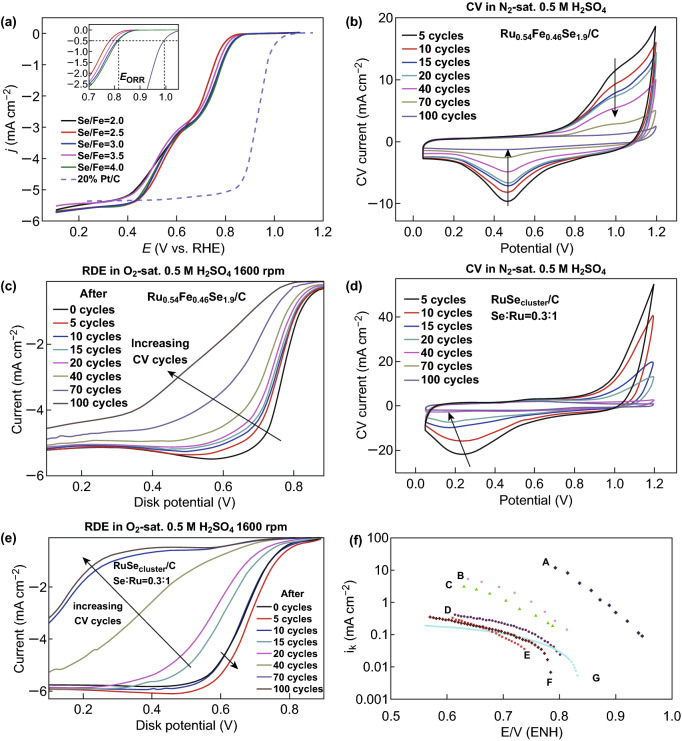
**a** RDE polarization curves of Pt/C (dashed line) and FeSe_2_/C (solid lines) prepared with different Se/Fe ratios in O_2_ saturated 0.1 mol L^−1^ KOH solution at 1600 rpm. **b** CV scan was subjected to electrocatalyst stability test at 50 mV sL^−1^. **c** Electrocatalyst stability tests O_2_ reduction current at 10 mV sL^−1^ and 1600 rpm was Ru_0.54_Fe_0.46_Se_1.9_/C. **d** CV scan was subjected to electrocatalyst stability test at 50 mV sL^−1^. **e** Electrocatalyst stability test O_2_ reduction current of RuSecluster/C (Se:Ru = 0.3:1) at 10 mV sL^−1^ and 1600 rpm. **f** ORR Tafel graphs. Adapted from Refs. [109–111] with permission. Copyrights: 2015, The Royal Society of Chemistry and 2009 (2010), Elsevier

Some researchers have fabricated a series of carbon-loaded FeSe_2_ nanoparticles by adjusting the iron-to-Se ratio, synthesized iron selenide-related compounds by the microwave method, and explored a series of their properties. Zheng et al. [109] used selenium dioxide (SeO_2_) and FeC_2_O_4_·2H_2_O as precursors to carry different molar ratios of Se/Fe on FeSe_2_ nanoparticles by the microwave method. Electrochemical experiments showed that when the Se-to-Fe ratio was changed from 2.0 to 4.0, the ORR potential changed from 0.781 to 0.814 V, and the electron transfer number changed from 3.3 to 3.9 (Fig. 12a). When FeSe_2_ is further reduced, Fe and Se are present on the surface of FeSe_2_/C. The degree of graphitization of the carbon support and the number of active sites of the ORR are weakly related to the Se/Fe ratio. However, in the current work, Ezeta et al. [110] used high-energy mechanical alloying to synthesize high-purity powders (Ru, Se, and Mo) into Ru–Mo–Fe and Ru–Se–Fe alloy nanoparticles. The Tafel parameter of the RDE results indicates that there is a primary ORR in both electrocatalytic systems to form water owing to a 4e-global multielectron transfer (Fig. 12f). The results of the electrocatalysis indicate that mechanical alloying is advantageous for obtaining nanoparticle electrocatalysts with good ORR properties. However, some researchers have synthesized iron selenide-related compounds by hydrogen annealing of carbonyl precursors and conducted a series of investigations on their properties. Chiao et al. [111] synthesized a Ru_1−*x*_Fe_*x*_Se_*y*_/C (*x* = 0.0–0.46, *y* = 0.4–1.9) catalyst by the hydrogen annealing of a carbonyl precursor. Here, we explore three factors that influence the catalytic activity of Ru_1−*x*_Fe_*x*_Se_*y*_/C. The substitution of metal Fe has the advantages of reducing the material cost, enhancing the activity of ORR, and increasing the yield of H_2_O_2_. The stability test results of Ru_0.54_Fe_0.46_Se_1.9_/C and RuSe_2.0_/C were obtained. The former had a higher activity decay rate than that of the latter (Fig. 12b–e). However, both catalysts had higher durabilities than that of the RuSecluster/C (Ru: Se = 1:0.3) agglomerated catalyst without annealing. By comparing the activity of the product, peroxide yield, and stability, the Ru_0.54_Fe_0.46_Se_1.9_/C catalyst can be used as an effective ORR catalyst in a fuel cell.

### Hydrogen Evolution Reaction

#### Cobalt Selenium

**Fig. 13:**
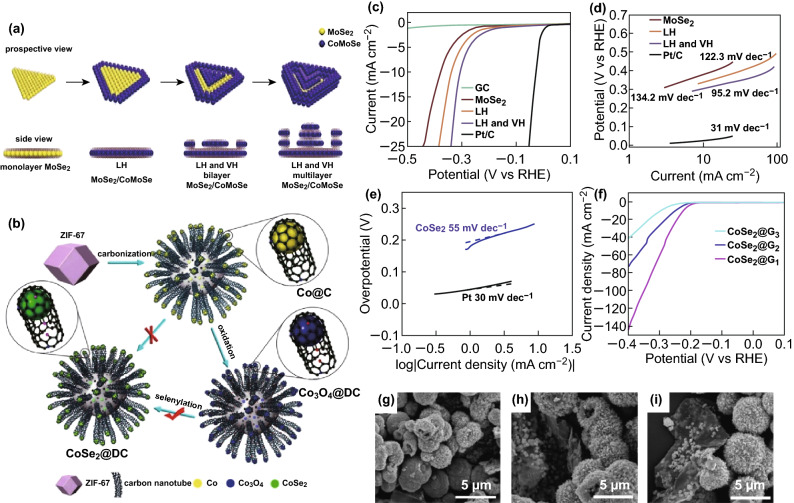
**a** Schematic representation of the formation of defective 2D platform MoSe_2_/CoMoSe LH and LH and VH nanomolecules. **b** Schematic diagram of the synthesis process of CoSe_2_@DC. **c** Electrochemical catalytic performance of 2D triangular MoSe_2_ monolayers, defective 2D platform MoSe_2_/CoMoSe LH, LH, and VH nanofilms on gas chromatographic electrodes. Polarization curve after infrared correction. **d** Electrochemical catalytic performance of 2D triangular MoSe_2_ monolayers, defective 2D platform MoSe_2_/CoMoSe LH, LH and VH nanofilms on gas chromatographic electrodes. Tafel plot. **e** Corresponding Tafel slope of CoSe_2_ and Pt. **f** Polarization curves of CoSe_2_@G_1_, CoSe_2_@G_2_, and CoSe_2_@G_3_. **g** FE-SEM image of CoSe_2_@G_1_. **h** FE-SEM image of CoSe_2_@G_2_. **i** FE-SEM image of CoSe_2_@G_3_. Adapted from [112–114] with permission. Copyrights: 2017, The Royal Society of Chemistry and 2016 (2017), Elsevier

It is well known that it is very difficult to increase the activity and stability of an electrocatalyst in the HER. Dai et al. [112] reported a facile method for synthesizing CoSe_2_ hollow microspheres using organic Se sources. Because the surface of CoSe_2_ with a hollow structure is rough, it is easy to expose the material to a more active site, thereby achieving good HER activity (the Tafel slope is 55 mV dec^−1^) (Fig. 13e). Because the hollow structure promotes the activity of the electrocatalyst, researchers hope to further improve the catalytic activity of the CoSe_2_ electrocatalyst by changing the conditions without changing the hollow structure. Additionally, it is clear that the addition of graphene does not change the hollow structure (Fig. 13g–i). The addition of graphene can significantly improve the HER activity of CoSe_2_; however, the increase in the graphene content reduces the performance of the catalyst (Fig. 13f). The excellent HER activity of CoSe_2_@G is attributed to the critical structure–function coupling effect. In addition, CoSe_2_@G nanocomposites and CoSe_2_ have high stability and durability, and because of their unique structure, they are expected to be widely used in the fields of optics, catalysis, sensors, and electronics. For the first time, Chen et al. [113] proposed a lateral heterostructure (LH) and a defective 2D stepped MoSe_2_/CoMoSe by adding an iso-doped Co to a MoSe_2_ nanofilm under high-temperature conditions, which is known as the method of double layering of hair. LH and vertical heterostructure (VH) nanolayers of MoSe_2_/CoMoSe are shown in Fig. 13a. MoSe_2_/CoMoSe LH and VH have an overpotential of 305 mV at a minimum current density of 10 mA cm^−2^, indicating that the HER activity is optimal (Fig. 13c). MoSe_2_/CoMoSe LH and VH have a minimum Tafel slope of − 95.2 mV dec^−1^ (Fig. 13d). This method is a simple and reliable technique for doping other metals into the 2D TMDCS atomic layer to create new alloy nanolayers to obtain new LHs or VHs on different substrates. Let us now consider the next generation of HER electrocatalysts. HER electrocatalysts with low overpotential and robust stability are among the most daunting challenges in energy conversion. Zhou et al. [114] synthesized CoSe_2_ nanoparticles embedded in defective carbon nanotubes (CoSe_2_@DC) by a three-step carbonation–oxidation–selenization reaction. The specific synthesis process is shown in Fig. 13b. CoSe_2_ nanoparticles, as a core of CoSe_2_@DC, partially embedded in defective carbon nanotubes, exhibit excellent HER activity, with a low initial potential of ~40 mV for RHE, a high current density (132 mV, 10 mA cm^−2^), and Tafel slope stability. The metal@carbon preoxidation strategy reported herein can expose the active sites of the core while maintaining the porous carbon skeleton, which has greater potential for application in electrocatalytic reactions.

#### Iron Selenium

Although there have been relatively fewer studies on iron selenide in HER, some researchers have recently synthesized and characterized a series of compounds related to iron selenide. Chang et al. [115] used a simple solvothermal method to synthesize 3D FeSe_2_ microfluid. The Se powder and FeCl_2_·4H_2_O were used as the raw materials and ethanolamine as the solvent. The band gap of the product and its photoluminescence properties are discussed. The results show that this sample exhibits excellent catalytic activity, indicating that these systems can be enriched in the HER catalyst family. The FeSe_2_ microfluid is a substance composed of nanosheets and has a thickness of 50–80 nm. The characteristic peak of the Se–Se vibration mode is shown on the Raman spectrum. The optical band gap was 1.48 eV when measured by the UV–visible absorption spectroscopy. The researchers also explored the catalytic activity of FeSe_2_ microfluids for hydrogen evolution and its photoluminescence properties. Finally, the possible growth mechanism of FeSe_2_ microfluids is proposed.

#### Bimetallic Selenium

Some researchers have explored the catalytic performance of HER by synthesizing ultrathin nanosheets of bimetallic selenide. Xiao et al. [116] first reported the synthesis of microspheres prepared from cobalt–iron–selenium ultrathin nanosheets as an electrocatalyst to HER. Because the prepared cobalt–iron–selenium ultrathin nanosheet microspheres have a high specific surface area, the cobalt–iron–selenium ternary compound exhibits a high HER electrocatalytic activity and low initial potential in an acidic medium. The presence of multiwalled CNTs improves the immobilization effect of the synergistic iron–selenium catalyst and the electron transfer between the catalyst and the electrode surface, thereby improving the stability and HER electrocatalysis. Nonprecious metal-based ferrous Se ternary compounds obtained from the rich elements of the earth can provide insights into new ways to explore hydrogen’s cost-effective catalysts for hydrogen energy development.

### Oxygen Evolution Reaction

The characteristics of inorganic nanowire arrays make them great prospects for the next generation of energy conversion and storage devices. Therefore, improved understanding of the nanowire growth mechanism is of significance for the expansion of nanowire applications. Du et al. [117] demonstrated the solvent thermal synthesis of Ni_3_Se_4_ and (Ni, Fe)_3_Se_4_ and the morphology of ultrathin nanometer tablets with efficient oxygen precipitation. The (Ni, Fe)_3_Se_4_-based solution has a small Tafel slope of 41 mV dec^−1^ at 25 mV, 10 mA cm^−2^. This is the first study on Ni_3_Se_4_-based double transition metal materials. As a catalyst for OER, it has an ultrathin nanosheet morphology and remarkable electrocatalytic activity, which is very promising for achieving high efficiency and long-term stability. Yu et al. [118] produced 3D mesoporous Ni_0.76_Fe_0.24_Se on NF by two successive hydrothermal methods. The prepared Ni_0.76_Fe_0.24_Se has an excellent superpotential, Tafel slope, and excellent stability in the alkaline solution. The simple synthetic route proposed here could also inspire the development of other transition-metal selenides, sulfides, and phosphating materials for further applications. Rose et al. [119] synthesized a CoP–CoSe_2_|CoP metal phosphating and CoSe_2_ metal sulfide mixed catalyst to elucidate their active form. The material exhibited excellent OER activity when subjected to a low overpotential of 240 mV, current density of 10 mA cm^−2^, and Tafel slope of 46.6 mV dec^−1^. This research not only laid the foundation for the development of OER complex mixed catalysts but also revealed the real active species in TMC/TMP.

### OER and HER

Conversion of the electrical energy output from renewable sources into chemical fuels (i.e., oxygen and hydrogen) is a promising strategy for electrochemical conversion. Furthermore, the decomposition of total electrochemical water has received considerable attention from researchers. The development of low-cost and highly active electrocatalysts is a key challenge associated with overall water splitting. To date, however, only a few materials have been able to catalyze two reactions in the same electrolyte simultaneously. Hou et al. [120] synthesized a vertically oriented Co_0.85_Se nanosheet by in situ growth on an electrochemically stripped graphene foil and constructed the material into a 3D strongly coupled ternary hybrid electrode. The deposited NiFe-layered double hydroxide was then further processed by the hydrothermal method. The obtained 3D fractionated mixture had the advantages of high surface area, strong coupling effect of 156 m^2^ g^−1^, and excellent catalytic activity for OER. Overvoltage values of only 1.50 and 1.51 V were required to obtain current densities of 150 and 250 mA cm^−2^, respectively. These overpotentials are much lower than those reported for Ir/C catalysts and nonprecious metals.

### Others

**Fig. 14:**
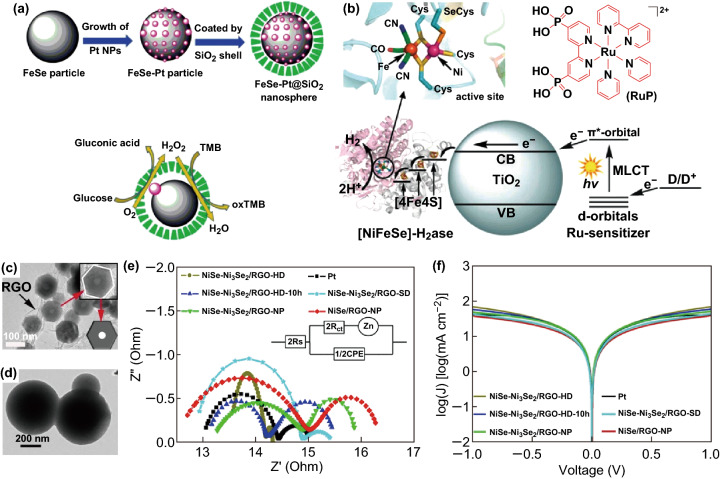
**a** Preliminary synthesis and enzyme cascade mechanism of FeSe-Pt@SiO_2_ nanohybrids. **b** Visible light drives H_2_ evolution of RuP–TiO_2_-H_2_ase. TEM image of **c** NiSe–Ni_3_Se_2_/RGO–HD and **d** FeSe–Pt@SiO_2_ microspheres. **e** Nyquist diagram of symmetrical elements made with NiSeNi_3_Se_2_/RGO series and Pt CE. **f** Tafel polarization curves of NiSeNi_3_Se_2_/RGO series and Pt CE symmetric cells I^−^/I^3−^. Adapted from Refs. [121, 124, 126] with permission. Copyrights: 2015 (2018), The Royal Society of Chemistry and 2018, American Chemical Society

Hollow and hybrid nanomaterials have novel electrocatalytic properties that make them excellent electrocatalysts compared to homogeneous solid nanostructures. Zhang et al. [121] synthesized a series of NiSe–Ni_3_Se_2_/RGO with hollow hexagonal nanodisk morphologies on graphene by controlling the heat treatment duration of the solvent. As shown in Fig. 14c, NiSe–Ni_3_Se_2_/RGO has a unique hollow and mixed structure. The electrocatalytic performance of NiSe–Ni_3_Se_2_/RGO with a hollow nanorod structure is better than that of NiSe/RGO and the solid NiSe–Ni_3_Se_2_/RGO nanostructures (Fig. 14f). Furthermore, as shown in Fig. 14e, the NiSe–Ni_3_Se_2_/RGO nanodisk with a hollow structure has a higher PCE and lower charge transfer resistance than Pt, and the electrochemical performance of NiSe–Ni_3_Se_2_/RGO is far better than that of other Pt-based devices. Xu et al. [122] synthesized a series of hexagonal nickel selenide nanowires by solution–liquid–solid (SLS) processing using nonmetallic molecular crystals as catalysts and successfully introduced SLS into the conventional low-temperature solution field. NiSe nanowire arrays were used as catalysts for electrochemical water oxidation as proof-of-concept applications. The experimental results show that NiSe nanowire arrays exhibit excellent stability and high activity. Kim et al. [123] investigated the redox chemistry of Se on magnetite under environmentally relevant *E*_H_ and *p*H conditions (+ 0.85 to approximately 1.0 V vs. Ag/AgCl; *p*H 4.0–9.5). The Se redox peak was found by a CV experiment at *p*H 4.0–8.0. A reduction peak of width approximately 0.5 V indicates a multielectron transfer process of a disproportionation reaction between selenite and Se(0) and Se(-II) and Se(-II) and selenium(IV). In chronoamperometry, when potential *P* is greater than or equal to − 0.6 V, the current–time transient shows a good linear relationship on the log–log scale. However, when the negative potential is larger, a deviation from the linear tendency is observed. The above results indicate that the nucleation and growth of Se(0) on the surface of magnetite can be explained by a progressive nucleation model. Some researchers have synthesized iron-related selenides by a three-step hydrothermal method and carried out a series of characterizations to explore their applications in electrocatalysis. Joe et al. [124] synthesized FeSe–Pt@SiO_2_ nanosphere nanoreactor by a simple three-step assembly method (Fig. 14a). An analysis of the microstructure of FeSe–Pt@SiO_2_ shows that it is composed of regular spheres with smooth surfaces (Fig. 14d). The study of its materials shows that FeSe–Pt@SiO_2_ nanospheres have both intrinsic glucose oxidase (GO^*x*−^) and peroxidase mimetic activity. Therefore, the researchers designed high stability based on nanostructures and a highly active artificial enzyme cascade system. It is worth noting that FeSe–Pt@SiO_2_ nanospheres can act as powerful nanoreactors to catalyze the self-organizing cascade. The high detection sensitivities of 0.227 and 1.136 nm were used to detect the color of FeSe–Pt@SiO_2_ nanospheres owing to their performance. FeSe–Pt@SiO_2_ nanospheres are expected to be applied as an enzyme mimetic in the field of diagnostics and biotechnology. Li et al. [125] synthesized a univalent Ni–Co–Se alloy-controlled hollow microsphere by a one-step hydrothermal method and used it as a CE material for DSSCs, which has excellent electrocatalytic activity. Ni–Co–Se-180 CE has the highest current density, lowest overpotential, and smallest charge transfer resistance compared with those of the other CE and Pt CE. Batteries with Ni–Co–Se-160, Ni–Co–Se-180, and Ni–Co–Se-200-based CEs achieved high efficiencies of 8.39%, 9.04%, and 8.72%, respectively, which are all higher than that of Pt CE (8.07%). This study shows that ternary Ni–Co–Se-based alloys can be used as low-cost and high-efficient CEs in DSSCs.

Some scientists have explored the synthetic structure model of the [NiFeSe] hydrogenase active site and conducted a series of investigations on photocatalytic hydrogen production. Recently, Erwin et al. [126] reported that D. baculatum [NiFeSe]–H_2_ase adsorbed on TiO_2_ nanoparticles has an unusual electrocatalytic stability. As proof of its applicability and concept, we assembled a photocatalytic H_2_ production system driven by visible light consisting of a hydrogenase directly adsorbed on dye-sensitized TiO_2_ nanoparticles. The catalytic process of visible light is shown in Fig. 14b. The useful properties of [NiFeSe]–H_2_ase from D. baculatum, especially its stability to TiO_2_ and some O_2_ tolerance to H_2_, should allow us to extend our work to include biohydrolysis water oxidation catalysts. Prior to this, Wombwell et al. [127] reported a synthetic structural model for the [NiFeSe] hydrogenase active site. Comparing [NiFe(‘S_2_Se_2_’)(CO)_3_] with previously reported thiolate analogs, the results show the selenate group in [NiFe(‘S_2_Se_2_’)(CO)_3_]. In the infrared light, the lower carbonyl stretching frequency is not produced in the spectrum. Electrochemical studies on [NiFe(‘S_2_Se_2_’)(CO)_3_] and [NiFe(‘S_4_’)(CO)_3_] show that neither of these two complexes is a homogeneous H_2_ precipitation catalyst, but rather a solid deposit is produced on the surface of the electrode for H_2_ precipitation catalysis in organic and aqueous solutions.

### Zn–Air Batteries

Recently, significant progress has been made in high-performance electrocatalysts. Therefore, metal–air electric devices and hydraulic decomposition devices will become electrocatalysts for efficient clean energy technology in OER, ORR, HER, or OER rechargeable metal–air batteries. They play a key role in water decomposition to reduce overpotentials and accelerate reaction kinetics [128–131]. Metallic selenides (M_*x*_Se, M = Co, Ni, and Fe) have inherent conductive metal properties that ensure efficient electron transfer during electrocatalysis. Therefore, the catalytic properties of transition-metal oxides and transition-metal sulfides are more effective than those of the sulfides [132–136]. Zheng et al. [137] synthesized Ni_*x*_Se (0.5 ≤ *x* ≤ 1) nanocrystals with different crystal compositions by using rapid environmental-pressure thermal injection. Among the series of synthesized Ni_*x*_Se, cubic Ni_0.5_Se has excellent OER catalytic activity. However, Ni_0.75_Se exhibits better HER and ORR properties and higher catalytic durability than that of noble metal catalysts in alkaline media. Owing to the excellent electrocatalytic performance of the half-reaction, the Ni_0.75_Se and Ni_0.5_Se electrodes achieved further assembly of the rechargeable zinc–air battery and overall water decomposition. The electrochemical tests showed low overpotential and high cycle efficiency and stability.

### Summary

By looking at the data in Table 3, we can compare the performances of iron, cobalt, and nickel selenium in electrocatalysis. By summarizing the above-mentioned articles on electrocatalysis, we can see that the current research on catalysts mainly focuses on ultrathin nanosheets. However, some scholars have also conducted research on the 3D nanoarray structure and have achieved certain results in this field. The 3D structured nanoarray can yield a larger number of active sites for the material, thereby increasing the catalytic activity of the electrocatalyst. We can also summarize the main ways to improve the electrocatalytic activity, as follows: (1) increase the number of active sites, design nanostructured catalysts, and increase the contact area of the electrolyte. (2) A good catalyst support is prepared, such as preparing a nanoarray on C and N. This review demonstrates that the activity of the electrocatalyst can be improved by these two methods.

**Table 3:** Application of iron/cobalt/nickel selenium in electrocatalysis

Materials	Testing condition	*E*_onset_ (V)^a^	*E*_j_ = 10 (V)^b^	Tafel slope (mV dec^−1^)	References
Co_0.85_Se nanosheets	0.1 M KOH	–	–	–	[104]
CoSe_2_/N-C	0.5 M H_2_SO_4_	–	–	–	[105]
[Co_4_(CO)_12_]	0.5 M H_2_SO_4_	–	0.79	− 120	[106]
CoSe	0.5 M H_2_SO_4_	0.5–0.6	0.3–0.55	–	[107]
FeSe_2_/C	0.1 M KOH	–	–		[109]
Ru–Se–Fe	0.5 M H_2_SO_4_	–	–	77	[110]
Ru_1−*x*_Fe_*x*_Se_*y*_/C	0.5 M H_2_SO_4_	–	–	–	[111]
CoSe_2_	0.5 M H_2_SO_4_	− 0.17	0.21	42	[112]
MoSe_2_/CoMoSe	0.5 M H_2_SO_4_	–	0.305	95.2	[113]
CoSe_2_@DC	0.5 M H_2_SO_4_	0.04	0.2	82	[114]
Co-Fe-Se	0.5 M H_2_SO_4_	–	–	–	[116]
(Ni,Fe)_3_Se_4_	1.0 M KOH	–	225	41	[117]
NiFeSe	1 M KOH	–	400	56	[118]
CoSe_2_|CoP	1 m KOH	0.269	240	46.6	[119]
(Co_0.85_Se) nanosheets	5 M H_2_SO_4_	–	0.26	47	[120]
Fe/Ni_2.4_/Co_0.4_-MIL-53	1.0 M KOH	–	–	–	[122]
FeSe	0.5 M H_2_SO_4_	–	–	–	[123]
FeSe-Pt@SiO_2_	0.5 M H_2_SO_4_	–	1.47	–	[124]
[NiFeSe]	0.5 M H_2_SO_4_	–	–	–	[126]
[NiFe(‘S_2_Se_2_’)(CO)_3_]	0.5 M H_2_SO_4_	–	–	–	[127]
FeSe_2_	0.5 M H_2_SO_4_	1.39	–	89	[115]
NiSe-Ni_3_Se_2_/RGO	0.5 M H_2_SO_4_	–	–	–	[121]
Ni_*x*_Se	5 M H_2_SO_4_	–	–	–	[138]

^a^*E*_onset_ for onset potential (V vs. RHE)

^b^*E*_j_ = 10 for overpotential required for the current density of 10 mA cm^−2^ (V vs. RHE)

## Conclusions and Future Outlook

Electrode materials play a vital role in the field of electrochemical energy storage and conversion. Therefore, many electrode materials have been explored and developed to improve the energy storage performance and conversion efficiency. This exploration process not only lays a foundation for the storage and transformation mechanism of electrochemical energy but also points out important standards for good electrode materials. Obviously, the research on M_*x*_Se_*y*_ (M = Fe, Co, Ni) and their composite has made significant progress. The ultrasmall nanostructure and ordered 3D structure of transition-metal selenides and their composite materials effectively improve the storage performance of electrochemical energy. M_*x*_Se_*y*_ (M = Fe, Co, Ni) are rich in electronic states and good electrical conductivity and have also been reported as active materials for energy storage.

However, the study of transition-metal selenides and their composites for the future development process still face enormous challenges. First, the electron conductivity and transition kinetics of transition-metal selenides and their complexes are low. We can improve the poor electron conductivity and buffer volume by designing selenides with suitable microstructures. Second, hollow/porous structural materials have yielded large surface areas, and their preparation requirements are so stringent that the fabrication of such porous materials is fraught with challenges. To overcome this problem, we can synthesize nanoparticles to increase the contact area between the electrolyte and the material. A certain oxide layer may form on the surface of the material, thus affecting the reversible capacity. Li et al. [58] controlled the surface oxide by controlling the selenization process, and the prepared FeSe_2_ nanorod/graphene component produced almost no surface oxide, thereby exhibiting excellent electrochemical performance.

Finally, the importance of M_*x*_Se_*y*_ (M = Fe, Co, Ni) in electrochemical energy storage and conversion is increasing. In the future, we can attempt to explore the effects of multielement transition-metal selenides on electrochemical energy storage and conversion. In addition, these could be applied to other types of batteries (Li–S batteries, Li–O_2_ batteries, Li–Se batteries, and zinc Na–Se). The application performance of batteries can be studied. Despite challenges, the rapid developments in research on these materials have paved the way for further development of these new functional materials.
